# Efficient luminescent stable Chichibabin diradicaloid for near-infrared imaging and photothermal therapy

**DOI:** 10.1038/s41377-025-01993-w

**Published:** 2025-08-26

**Authors:** Ting Liu, Zihao Zhu, Shengjie Wang, Li Shen, Alim Abdurahman, Xiaomin Liu, Geyu Lu

**Affiliations:** 1 State Key Laboratory of Integrated Optoelectronics, JLU Region, College of Electronic Science and Engineering, Jilin University, Qianjin Avenue 2699, Changchun, 130012 China; 2 College of Chemical Engineering and Environmental Chemistry, Weifang University, Weifang, 261061 China

**Keywords:** Biomaterials, Applied optics

## Abstract

Diradicaloids have garnered significant attention due to their unique electronic, photophysical properties and potential applications in functional materials. Characterized by a narrow band gap and near-infrared (NIR) absorption, diradicaloids are promising candidates for NIR-guided photothermal therapy. However, they are often unstable and exhibit non-emissive properties due to high chemical reactivity and very efficient internal conversion. Herein, we report a remarkably stable Chichibabin diradicaloid, TT-CzPh, which exhibits NIR luminescence (λ_em_ = 821 nm) with an efficient photoluminescence quantum yield (PLQY) of 6.4%. Surprisingly, TT-CzPh not only exhibits excellent NIR imaging but also has a very high photothermal conversion efficiency (PCE) of 87.5%. In vivo experiments with TT-CzPh nanoparticles demonstrated their effectiveness in tumor photoablation guided by NIR imaging. This work not only advances the development of stable, efficient luminescent Chichibabin’s hydrocarbons but also opens new avenues for bioimaging and cancer phototherapy applications.

## Abstract (web summary)

An efficient, stable, and luminescent Chichibabin diradicaloid, TT-CzPh, boasts a notably high photoluminescence quantum yield (PLQY) of 6.4%. It exhibits extremely bright near-infrared (NIR) imaging capability and an exceptionally high photothermal conversion efficiency (PCE) of 87.5%.

## Graphical abstract

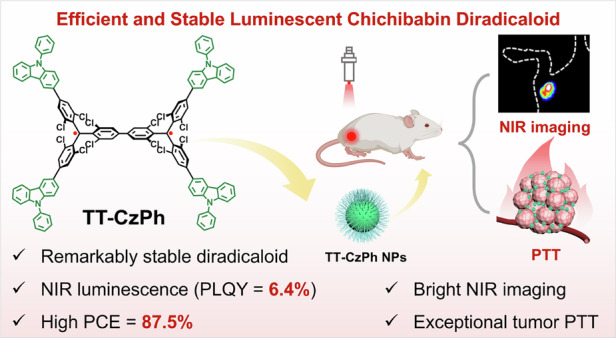

## Introduction

Diradicaloids^1–4^ have attracted considerable interest due to their synthetic complexity^5^, unique bonding^6^, intriguing physical properties^7–9^, and potential applications in functional materials^10–15^. A prominent example is Chichibabin’s hydrocarbon, first synthesized by Tschitschibabin in 1907^16^, which has been studied for over a century^17–27^. However, its intrinsic instability has hindered further exploration and practical applications. Consequently, extensive research has focused on developing more stable derivatives and analogs^28,29^. Thanks to their strong spin-coupling effects, these molecules often exhibit narrow band gaps and remarkable light absorption^30,31^, particularly in the near-infrared (NIR) region^24,26,27,32–37^, making them ideal candidates as photothermal agents^38–40^. Notably, Wang’s group recently reported highly stable NIR luminescent diradicaloids used as phototheranostic agents^41^. Furthermore, Zeng’s team reported three stable diradicaloids derived from Chichibabin’s hydrocarbon that feature intramolecular charge transfer (ICT) characteristics. One of them has been applied in tumor photothermal therapy, demonstrating their great potential for biomedical applications^42^. In parallel, Yan’s group^43^ has extended the application of luminescent Thiele’s hydrocarbon^32,33^ into the biomedical realm, specifically exploring its potential in photodynamic therapy. These advancements highlight the growing momentum of organic radical theranostics, which are drawing considerable attention for their promise in advancing precision medicine.

**Fig. 1:**
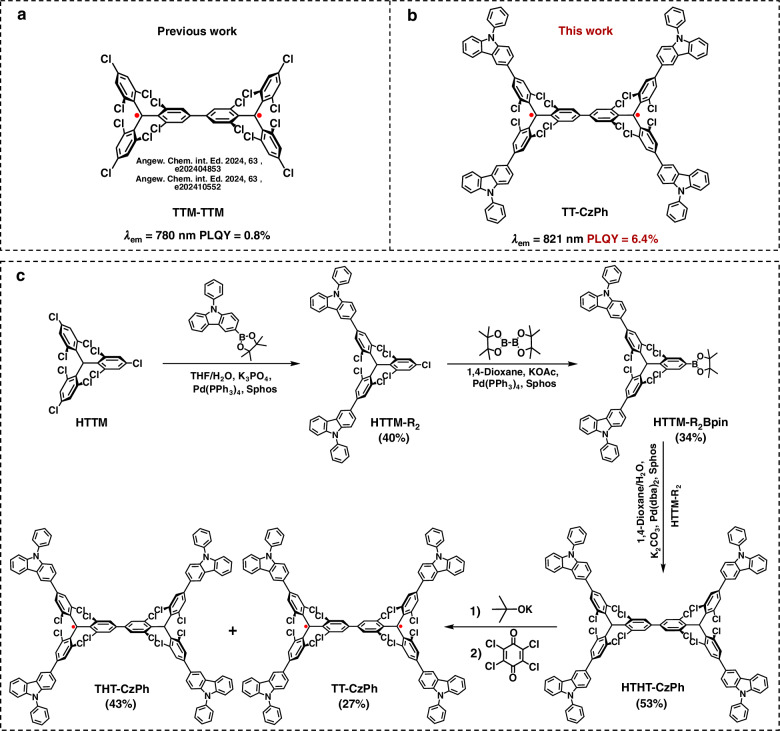
Strategy for diradicaloids design. **a** Structure of the previously reported Chichibabin’s hydrocarbon, TTM-TTM. **b** Structure of the newly synthesized Chichibabin’s hydrocarbon, TT-CzPh. **c** Synthetic routes are toward TT-CzPh

Recently, Delius’ group and our team reported the first chlorine-substituted luminescent Chichibabin’s hydrocarbon, TTM-TTM^26,27^, which exhibits exceptional stability due to effective steric hindrance and spin delocalization. Notably, the emission of TTM-TTM (Fig. 1a) reaches the near-infrared region (λ_em_ = 780 nm). However, its small energy gap and strong spin-coupling enhance the Franck–Condon factors^44^, increasing internal conversion^45,46^ and limiting its photoluminescence quantum yield (PLQY) to only 0.8%. This low PLQY restricts its potential for optical applications. We hypothesize that, with improved molecular design to enhance PLQY, these molecules could offer superior properties for NIR imaging and photothermal therapy, opening doors to their applications in cancer phototheranostics.

In this work, we have successfully designed and synthesized a Chichibabin’s hydrocarbon derivative, TT-CzPh (λ_em_ = 821 nm) (Fig. 1b), with significantly improved luminescence. By breaking the alternating symmetry and modulating donor conjugation, we enhanced the transition oscillator strength, resulting in superior photophysical properties. TT-CzPh exhibits a redshifted absorption spectrum, with a ~25 nm shift in the long absorption band associated with luminescence, compared to TTM-TTM^26,27^. The molecule’s extended outer structure not only improves its photostability but also boosts its PLQY to 6.4%. Moreover, TT-CzPh exhibits extremely bright NIR imaging and an excellent photothermal conversion efficiency (PCE) of 87.5%. For enhanced biocompatibility, we assembled TT-CzPh into water-soluble nanoparticles (NPs), which still showed prominent NIR imaging capabilities both in vitro and in vivo. In vivo studies further confirmed the exceptional photothermal tumor ablation efficacy of TT-CzPh NPs in a 4T1 tumor-bearing mouse model. This study not only provides valuable insights into the development of stable, efficient luminescent Chichibabin’s hydrocarbons but also opens new possibilities for bioimaging and cancer phototherapy applications.

## Results

### Synthesis and structural characterization of TT-CzPh

**Fig. 2:**
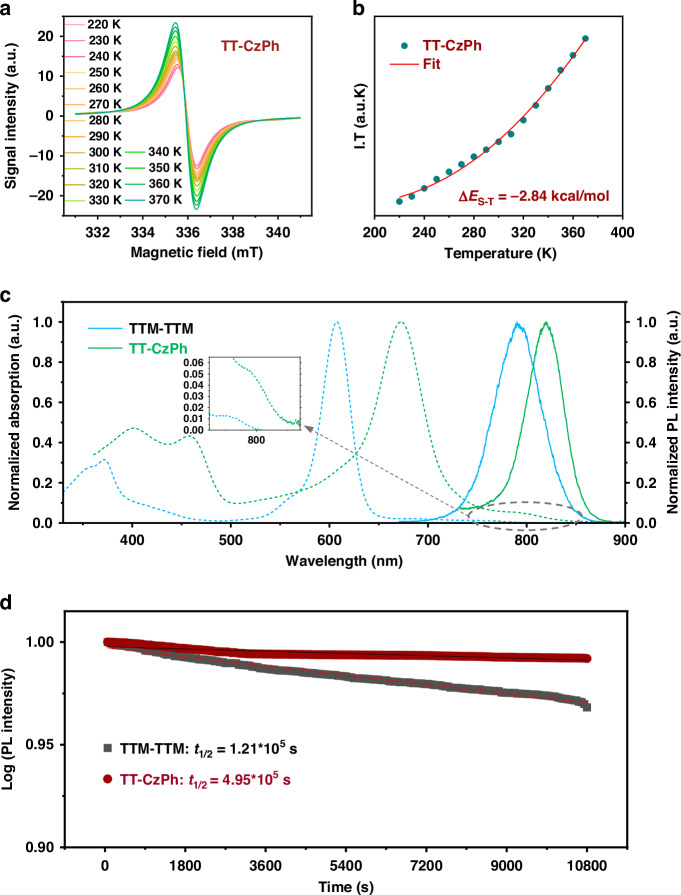
The properties of diradicals. **a** VT-EPR spectra of powder samples of TT-CzPh from 220 to 370 K. **b** Fitting curves of the corresponding ESR spectra according to the Bleaney-Bowers equation. **c** Normalized absorption (blue dot) and PL spectra (blue line) of TTM-TTM in toluene (10^−5^ M) and normalized absorption (green dot) and PL spectra (green line) of TT-CzPh in toluene (10^−5^ M) at room temperature. **d** Plots showing the PL intensity decay of radicals in toluene under 375 nm continuous photoexcitation (emission intensities are normalized)

The recently reported TTM-TTM molecule (Fig. 1a) achieved luminescence and significantly enhanced stability through the selective chlorination of hydrogen atoms in the original Chichibabin’s hydrocarbon framework^26,27^. It is worth considering whether replacing the four peripheral chlorine atoms of TTM-TTM with mild donors could further improve its photophysical properties. Based on this hypothesis, we skillfully incorporated 3-substituted-9-phenyl-9H-carbazole (3PCz) into the skeleton of TTM-TTM, resulting in a novel Chichibabin’s hydrocarbon derivative, TT-CzPh (Fig. 1b), which exhibits significantly improved luminescence. The detailed synthesis procedures (Fig. 1c) are provided in the Supplementary Information S1. ^1^H nuclear magnetic resonance (NMR) spectra, ^13^C NMR spectra, and high-resolution matrix-assisted laser desorption/ionization time-of-flight mass spectrometry (HR-MALDI-TOF-MS) were used to characterize the precursor molecule HTHT-CzPh (Figs. S7–S10). Subsequently, HR-MALDI-TOF-MS, high-performance liquid chromatography (HPLC), and elemental analysis were used to characterize TT-CzPh (Figs. S14–S16). The powder of TT-CzPh exhibits a single-line electron paramagnetic resonance (EPR) signal in the Δ*m*_s_ = ±1 region, with an additional signal corresponding to the Δ*m*_s_ = ±2 transitions observed at 295 K, confirming its diradical nature (Fig. S17). In variable-temperature (VT) EPR measurements (220–370 K), the signal intensity of TT-CzPh powder gradually decreases as the temperature lowers (Fig. 2a), corroborating the presence of an open-shell singlet (OS) ground state. Furthermore, fitting the EPR curves with the Bleaney-Bowers equation^47^ (Fig. 2b) estimates the singlet-triplet energy gap (Δ*E*_S-T_) of TT-CzPh to be −2.84 kcal/mol, which is consistent with theoretical calculations (Table S3) and slightly larger than TTM-TTM. At the theoretical level of PUB3LYP/6-31 G (d, p), Yamaguchi’s equation was used to calculate the diradical character (*y*_0_) of TT-CzPh, which is 0.72 (Table S3), similar to TTM-TTM. This high value reflects its open-shell characteristic.

### Photophysical properties

After confirming the structure of TT-CzPh, we further investigated its photophysical properties. In toluene solution, the UV/Vis absorption spectra and photoluminescence (PL) spectra of TT-CzPh exhibit a significant bathochromic shift (Fig. 2c). Specifically, both the main and long absorption bands of TT-CzPh show a redshift, exceeding 25 nm. Correspondingly, the emission spectra of TT-CzPh (λ_em_ = 822 nm) demonstrate a notable redshift, which is a 42 nm shift compared to TTM-TTM. Unexpectedly, the PLQY of TT-CzPh has increased by 6.5 times (Table S1). The enhancement of luminescence efficiency is even more pronounced in PMMA films, where the PLQY reaches 6.4%. In addition, Table S1 compares the photophysical properties of the diradical TT-CzPh and the monoradical THT-CzPh. Their UV/Vis absorption spectra and PL spectra differ significantly (Fig. S22). The wide absorption peak of TT-CzPh can reach the NIR region, and the emission peak is also located in the NIR region, which provides the possibility for in vivo imaging. In contrast, the monoradical THT-CzPh doesn’t have this advantage. The insets of Fig. 2c and Fig. S26 further demonstrate that the molar absorptivity (*ε*) of TT-CzPh at the long absorption band is significantly higher than that of TTM-TTM at the corresponding absorption band, which may account for the increase in PLQY. Furthermore, we performed time-dependent density functional theory (TD-DFT) calculations. According to the theoretical calculation results (Fig. S27 and Table S4), the absorption band at 785 nm of TT-CzPh corresponds to the electronic transition from β-HOMO to β-SOMO, which is the key absorption associated with the emission. The oscillator strength of TT-CzPh at the long absorption band is also markedly higher than that of TTM-TTM, which is consistent with the observation results of the UV/Vis absorption spectra.

To gain a deeper understanding of these phenomena, hole-electron analysis was carried out to interpret the electronic excited state characteristics of TT-CzPh using the Multiwfn program^48–50^. As a result, due to the introduction of peripheral donor groups, a certain degree of hole delocalization is observed in the TT-CzPh molecule, leading to partial separation of hole and electron (Fig. S28). In contrast, the hole and electron in TTM-TTM almost completely overlap, with a Sr value of 0.957 (Table S5). The heat map, based on the contribution of molecular fragments to the hole and electron, provides a clearer visualization of this phenomenon (Fig. S29). In general, when the degree of electron-hole separation is minimal, electrons are more likely to couple with high-frequency vibrational modes, which increases non-radiative losses^51^. On the other hand, overly strong electron-phonon coupling can cause the excitation energy to be released as heat through multi-phonon decay rather than as light, thereby reducing the PLQY of molecules^52^, implying that materials with such characteristics may have high PCE. In fact, TTM-TTM may be such a case, thus potentially exhibiting high PCE. In contrast, introducing the donor group 3PCz in TT-CzPh reduces electron-hole overlap, which not only reduces non-radiative losses but also enhances the oscillator strength (as shown in Table S5). It is the simultaneous occurrence of these two effects that contributes to the increased PLQY of TT-CzPh. In addition, thanks to the protective effect of the molecule’s outer extended structure, the photostability of TT-CzPh (Fig. 2d) is significantly enhanced compared to TTM-TTM, with an improvement of approximately 4 times. The optimization of these photophysical properties, particularly the redshifted near-infrared emission peak and higher PLQY, demonstrates the great potential of TT-CzPh for practical applications as a cancer phototherapeutic agent.

### Fluorescence imaging and photothermal performance

**Fig. 3:**
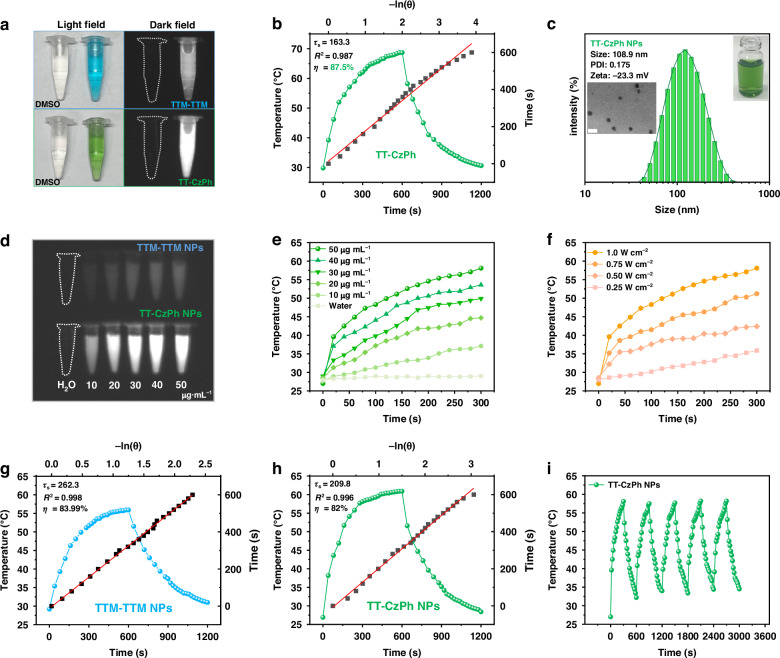
The photothermal properties of diradicals and their nanoparticles. **a** Smartphone-shot images of TTM-TTM and TT-CzPh in DMSO (concentration: 25 μg mL^−1^) (left) and the corresponding NIR fluorescence images captured with a 700 nm long-pass filter at excitation wavelength 655 nm (right). **b** Photothermal heating and cooling curves (linear fit-ln(θ)-cooling time) for TT-CzPh (Solvent: DMSO). **c** The particle size distributions of TT-CzPh NPs in aqueous solution were determined by DLS method. The insert is TEM image of TT-CzPh NPs, scale bar: 200 nm. **d** NIR fluorescence images captured with a 700 nm long-pass filter at excitation wavelength 655 nm of TTM-TTM NPs and TT-CzPh NPs at different concentrations. **e** Concentration-dependent temperature variation of TT-CzPh NPs in aqueous solution under 655 nm laser irradiation (1.0 W cm^−2^). **f** Power-density-dependent temperature variation of TT-CzPh NPs in aqueous solution (50 μg mL^−1^) under 655 nm laser irradiation. Photothermal heating and cooling curves of **g** TTM-TTM NPs and **h** TT-CzPh NPs with respect to the linear fitting −ln(θ) versus cooling time (50 μg mL^−1^, 655 nm, 1.0 W cm^−2^). **i** Photothermal stability of TT-CzPh NPs after five on/off cycles of 655 nm laser irradiation (1.0 W cm^−2^)

It is well established that excellent NIR imaging capabilities^53,54^ and high PCE^41,55–61^ are essential for effective photothermal agents. Therefore, the imaging and photothermal conversion abilities of the TT-CzPh were evaluated. We found that TT-CzPh exhibited a significantly brighter fluorescent signal in DMSO solution compared to TTM-TTM (Fig. 3a), and further analysis revealed a high PCE of 87.5% (Fig. 3b). Additionally, TT-CzPh demonstrated a rapid warming rate and good photothermal stability (Figs. S31–S33), while monoradical THT-CzPh had no photothermal effect (Fig. S30). These superior NIR imaging capabilities and high PCE make TT-CzPh an ideal candidate for bio-photothermal therapy applications.

To enable biological applications, we prepared water-soluble NPs of TT-CzPh via self-assembled nanoprecipitation, utilizing the amphiphilic polymer DSPE-mPEG_2000_ as the encapsulation agent. Characterized by excellent biocompatibility and the ability to enhance tumor-targeting efficiency through the Enhanced Permeability and Retention (EPR) effect, DSPE-mPEG_2000_ has found extensive applications in tumor therapy, vaccine development, and related biomedical fields^62–67^. The TT-CzPh NPs exhibited a spherical structure with an average size of 108.9 nm and showed better homogeneity (Figs. 3c, S34, S35). Their negative charge facilitates passive targeting via the EPR effect, followed by internalization and accumulation into the lysosomes of tumor cells^68,69^. EPR spectra confirmed their diradical nature in water (Fig. S36). UV/Vis absorption spectra showed that TT-CzPh NPs possess a broad absorption peak and strong light absorption in the NIR region (Fig. S37). Notably, the ε of TT-CzPh NPs was 2.82 × 10^4^ M^−1^ cm^−1^, 6.17 times higher than that of TTM-TTM NPs at 655 nm. This was further supported by brighter NIR luminescence images of TT-CzPh NPs at varying concentrations (Figs. 3d and S38).

Further, the photothermal performance of TT-CzPh NPs was also investigated. The temperature changes in the aqueous solution were positively correlated with both the concentration of TT-CzPh NPs and the laser power density (Fig. 3e, f). TT-CzPh NPs exhibited a faster warming rate compared to TTM-TTM NPs (Fig. 3g, h), attributed to their higher molar absorptivity. The PCE of TT-CzPh NPs was calculated to be 82%, which combines the advantages of a high PCE and near-infrared luminescence compared to the organic diradical materials, organic molecules, as well as electrodeless photothermal materials that have been reported so far (Table S6).

Additionally, TT-CzPh NPs demonstrated excellent photothermal stability, maintaining performance across multiple heating-cooling cycles (Fig. 3i). The absorption spectra, EPR spectra, and particle sizes of TT-CzPh NPs remained unchanged before and after laser irradiation (Figs. S41, S42). The homogeneity and stability of TT-CzPh NPs were also preserved when stored at 4 °C or in various physiological environments (Figs. S43–S45). These findings highlight the superior NIR imaging capabilities, high photothermal efficiency, and robust physiological stability of TT-CzPh NPs, making them ideal candidates for NIR imaging and bio-photothermal tumor therapy.

### In vitro cytotoxicity against cancer cells

**Fig. 4:**
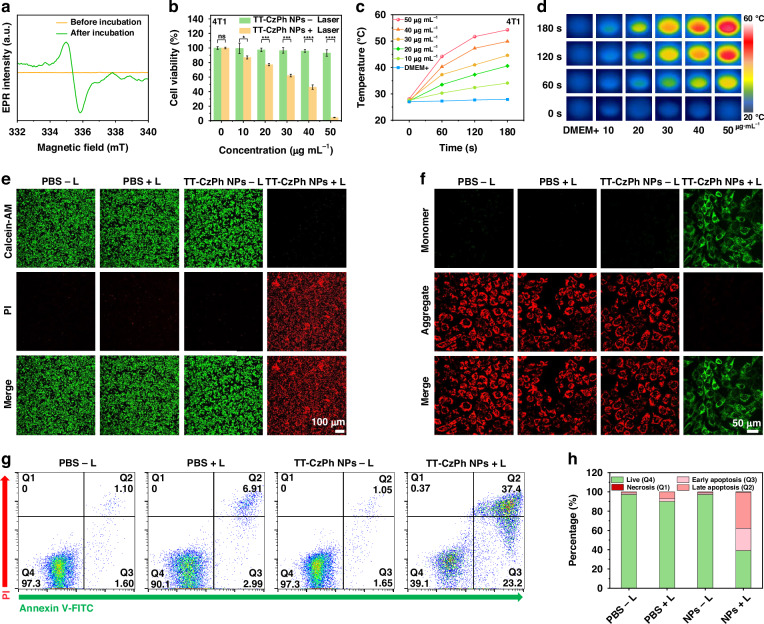
Cellular uptake and in vitro cytotoxicity of TT-CzPh NPs. **a** EPR signals of TT-CzPh NPs in cell suspensions before and after incubation with 4T1 cells. **b** Viabilities of 4T1 cells incubated with TT-CzPh NPs at various concentrations with/without laser irradiation (655 nm, 1.0 W cm^−2^, 3 min, *n* = 3, *P < 0.05, **P < 0.01, ***P < 0.001, ****P < 0.0001). **c** Temperature-time curves of different concentrations of TT-CzPh NPs and 4T1 cells after 4 h incubation. **d** Infrared thermal images of different concentrations of TT-CzPh NPs and 4T1 cells after 4 h incubation (655 nm, 1.0 W cm^−2^). **e** Live/dead co-staining images of 4T1 cells using Calcein-AM (green) and PI (red) assay, and **g** flow cytometry analysis using Annexin V-FITC/PI staining treated with different formulations. **f** JC-1 staining of 4T1 cells after being treated with PBS-L, PBS + L, TT-CzPh NPs-L, TT-CzPh NPs+L groups (‘-L’ without laser irradiation, ‘+L’ with laser irradiation). **h** Quantitatively analysis of live cells and the apoptosis degree of the results in (**g**)

Given their excellent photothermal properties, the in vitro antitumor effects of TT-CzPh NPs were first investigated. By utilizing the unique unpaired electronic characteristics of TT-CzPh NPs, we confirmed their uptake by cells by testing the EPR signals of cell suspensions before and after incubation with the NPs (Fig. 4a). The impact of TT-CzPh NPs on cell viability was then assessed. As shown in Fig. 4b, in the absence of laser irradiation, TT-CzPh NPs caused minimal damage to mouse breast cancer cells (4T1), indicating good biocompatibility. However, upon laser irradiation, cell viability dropped significantly from 100% to approximately 5% as the concentration of TT-CzPh NPs increased, demonstrating their strong photothermal effect. A similar photothermal ablation effect was observed in human hepatocellular carcinoma cells (HepG2), as shown in Fig. S46. Figure 4c shows the temperature increase in 4T1 cells under laser irradiation with varying concentrations of TT-CzPh NPs, with corresponding infrared images shown in Fig. 4d. At a concentration of 50 μg mL^−1^, the temperature rose to 54.3 °C within 3 min, confirming that TT-CzPh NPs effectively elevated the temperature to levels sufficient for cancer cell ablation.

To assess cell death visually, we performed live/dead co-staining using Calcein-AM/PI. As shown in Fig. 4e, cells treated with TT-CzPh NPs (50 μg mL^−1^) and laser alone exhibited strong green fluorescence, indicating no impact on cell viability. In contrast, after 5 min of laser irradiation, cells treated with TT-CzPh NPs showed nearly complete red fluorescence, with the red/green fluorescence ratio (F_R_/F_G_) increasing from 0.54 to 1.72 (Fig. S47). Flow cytometry analysis using the Annexin V-FITC/PI Apoptosis Detection Kit showed that the apoptosis rate in cells treated with TT-CzPh NPs of laser irradiation was approximately 60% (combining early and late apoptosis stages, Q2 and Q3), compared to less than 10% in the group treated with laser only (Fig. 4g, h). These results demonstrate the strong photothermal effects of TT-CzPh NPs on tumor cells in vitro upon laser irradiation. Mitochondrial membrane potential (MMP) analysis using the JC-1 probe confirmed that TT-CzPh NPs induced apoptosis after treatment (Fig. 4f). The Monomer-to-Aggregate fluorescence ratio (F_M_/F_A_) increased significantly from 0.10 to 4.53 (Fig. S48), indicating that the photothermal effect of TT-CzPh NPs caused mitochondrial dysfunction, leading to apoptosis and variations in MMP.

### In vivo photothermal therapy

**Fig. 5:**
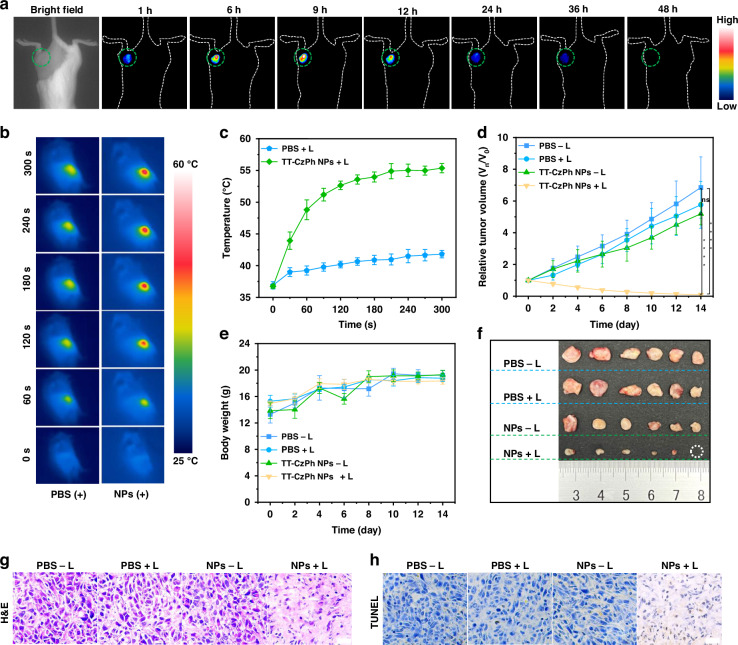
In vivo fluorescence imaging and photothermal therapy of TT-CzPh NPs. **a** NIR fluorescence imaging of tumors in living mice at different time points after TT-CzPh NPs injection. The tumor region is indicated with a green dashed circle. **b** Temperature changes at tumor sites with increasing time under continuous laser irradiation after treatment with PBS and TT-CzPh NPs. **c** Thermal images of mice 9 h postinjection of the TT-CzPh NPs aqueous solution under 655 nm laser irradiation (1.0 W cm^−2^) for 5 min (*n* = 6). **d** Average relative tumor volume (RTV) of mice after different formulations (*n* = 6; *P < 0.05, **P < 0.01, ***P < 0.001, ****P < 0.0001). **e** Average body weights of mice over a 14-day period for different treatment groups (*n* = 6). **f** Images of tumors extracted from 4T1 tumor-bearing mice ex vivo after 14 days of treatment. H&E (**g**) and TUNEL (**h**) staining of tumor tissues from mice sacrificed 14 days posttreatment, scale bar = 25 μm

Building on our previous explorations, we applied TT-CzPh NPs as photothermal agents for bio-photothermal tumor therapy. Initially, we conducted an in vivo fluorescence imaging study on a 4T1 tumor-bearing BALB/c mouse model to identify the optimal therapeutic window. Following the injection of TT-CzPh NPs, the fluorescence signal in the tumor region gradually intensified, peaking at 9 h and disappearing by 48 h, reflecting the metabolic processing of the NPs in vivo (Fig. 5a). Furthermore, TT-CzPh NPs demonstrated excellent fluorescence stability, with minimal variation in intensity over 48 h (Fig. S49). Based on these observations, the optimal treatment window was determined to be 9 h, with a subsequent dosing interval of 48 h. Furthermore, the main organs (heart, liver, spleen, lung, and kidney) of the experimental mice, along with tumor tissues, were collected 48 h after injection. At 48 h, no fluorescent signal of the nanoparticles was detected in the tumor (Fig. S50a, b). In addition, at 108 h, almost no signal of the nanoparticles was detected in the liver, which is the main metabolic organ (Fig. S50c), indicating that the nanoparticles have been metabolized and cleared.

Encouraged by the promising in vitro therapeutic performance, we evaluated the therapeutic efficacy of TT-CzPh NPs in a 4T1 tumor-bearing mouse model. Mice with tumors approximately 70 mm^3^ in volume were randomly assigned to our groups: PBS − L, PBS + L, TT-CzPh NPs − L, and TT-CzPh NPs + L. Tumor site temperature was monitored in real time during laser irradiation using a FLIR EX-Series camera (Fig. 5b). As shown in Fig. 5c, the temperature at the tumor site in the TT-CzPh NPs + L group rapidly increased from 36.2 °C to 52.5 °C within 2 min, stabilizing at around 55.0 °C, an effective temperature for tumor ablation. In contrast, minimal temperature change was observed in the PBS + L group. Over the 14-day treatment period, both tumor volume and body weight were monitored. No significant decrease in body weight was observed across all groups (Fig. 5e), while tumor volume in the TT-CzPh NPs + L group was significantly reduced (Figs. 5d, f, S51). These results provide strong evidence of the substantial photothermal tumor ablation effect of TT-CzPh NPs.

Additionally, hematoxylin and eosin (H&E) staining of major organs from mice treated revealed no significant physiological abnormalities compared to the other groups (Fig. S52). H&E staining of tumor tissues showed significant necrosis in the TT-CzPh NPs + L group, which was absent in the other groups (Fig. 5g). Terminal deoxynucleotidyl transferase (TdT)-mediated dUTP nick-end labeling (TUNEL) staining of excised tumors revealed the highest levels of apoptosis and necrosis, coupled with the lowest level of proliferation, in the TT-CzPh NPs + L group (Fig. 5h). These findings suggest that TT-CzPh NPs mediated photothermal therapy is highly effective and non-toxic to normal tissues. Finally, we systematically evaluated the biocompatibility of TT-CzPh NPs in vivo. The hemolysis rate of TT-CzPh NPs was as low as 3.4% when the concentration of TT-CzPh NPs was 100 μg mL^−1^ (Fig. S53), which indicated that TT-CzPh NPs have good biocompatibility.

## Discussion

In summary, we have successfully developed a stable, efficient luminescent Chichibabin diradicaloid TT-CzPh by employing the strategy of breaking the alternating symmetry and modulating donor conjugation. Specifically, the introduction of an extended outer layer structure endows TT-CzPh with excellent light stability and a redshifted NIR emission peak (λ_em_ = 821 nm), compared to TTM-TTM. The PLQY of TT-CzPh is significantly enhanced to 6.4%, and the phenomenon is further elucidated by quantum chemical computations. Additionally, TT-CzPh possesses extremely bright NIR imaging capabilities and an exceptionally high PCE of 87.5%. Based on this, we prepared water-soluble nanoparticles, TT-CzPh NPs, to explore biological applications. TT-CzPh NPs not only exhibit excellent NIR imaging performance but also achieve a PCE of up to 82%. Furthermore, their outstanding imaging capability effectively guided photothermal tumor therapy in vivo. To our knowledge, TT-CzPh is the first stable Chichibabin’s hydrocarbon that combines efficient luminescence and high PCE, successfully applied to bioimaging-guided tumor therapy. This study provides new insights into the development of efficient NIR luminescent Chichibabin’s hydrocarbons and expands the potential applications of luminescent diradicaloids in disease localization and cancer therapy.

## Materials and methods

### Materials

All chemical agents and solvents, unless otherwise stated, were purchased from commercial suppliers and used directly without further purification. Column chromatography was performed with silica gel (200–300 mesh). 1, 2-Distearoyl-sn-glycero-3-phosphoethanolamine-N-[methoxy (polyethylene glycol) - 2000] (DSPE-mPEG_2000_) was purchased from Aladdin. Calcein-AM/PI Dual Staining Kit, mitochondrial membrane potential assay kit (JC-1) by Beijing Solarbio Sciences & Technology Co., Ltd. DMEM medium, Fetal bovine serum (FBS), Trypsin, Penicillin, and Streptomycin were purchased from Dalian Meilun Biotechnology Co., Ltd.

### General characterization

The ^1^H nuclear magnetic resonance (NMR) spectra were recorded in deuterated dichloromethane (CD_2_Cl_2_) on a Bruker Avance-III 500 NMR spectrometer at ambient temperature. Matrix-assisted laser desorption/ionization time-of-flight (MALDI-TOF) mass spectra were recorded on a Bruker Autoflex speed TOF/TOF mass spectrometer with DCTB as a matrix. High-resolution MALDI-TOF mass spectra were measured with a Bruker Autoflex speed TOF spectrometer. High-performance liquid chromatography (HPLC) experiments were conducted on an Agilent 1260 Infinity II HPLC-MS equipped with an InfinityLab Poroshell 120. Electron paramagnetic resonance (EPR) spectra were recorded on a Bruker ELEXSYS-II E500 CW-EPR spectrometer. Ultraviolet-visible (UV-Vis) and photoluminescence (PL) spectra of the radicals were recorded on a Shimadzu UV-2550 spectrophotometer and a HITACHI F-4700 spectrophotometer. The PLQYs and the fluorescence lifetimes were measured with FLS980.

The size distribution and zeta potential were analyzed by dynamic light scattering (DLS) using a Zetasizer-Nano ZSZEN3600 apparatus (Malvern Instruments, UK). The nanoparticles (NPs) of particle size and morphology were determined using a JEM-2100 transmission electron microscope (TEM). The confocal cell images were obtained using a two-photon Nikon A1RMP microscope. Photothermal images and temperature variation were captured by a FLIR E8-XT camera (FLIR Systems). In vivo imaging and organ imaging were performed using an in vivo imaging chamber (SRAR-BIOGENII, JXXQ) provided by Changchun Jixian Xingqun Technology Co., Ltd.

### Synthesis of molecules and nanoparticles (NPs)

The detailed synthetic steps of molecules can be found in Supplementary Information S1. The synthesis of nanoparticles and the specific steps of in vivo and in vitro experiments can be found in the Supplementary Information S2 and S3.

## Supplementary information

Supplementary Information
