# Supplementary material for: Efficient luminescent stable Chichibabin diradicaloid for near-infrared imaging and photothermal therapy
Source: Light Sci Appl. 2025 Aug 26;14:289. doi: 10.1038/s41377-025-01993-w (PMC12381269; doi:10.1038/s41377-025-01993-w)
Supplement: Supplementary file 1 — Supplementary Information [file 41377_2025_1993_MOESM1_ESM.pdf]

Supplementary Information

Efficient Luminescent Stable Chichibabin Diradicaloid for Near-infrared Imaging and Photothermal Therapy

Ting Liu,†[a] Zihao Zhu,†[a], Shengjie Wang,[a] Li Shen,[b] Alim Abdurahman,*[a] Xiaomin Liu,*[a] Geyu Lu *[a]

[a] Ting Liu, Zihao Zhu, Shengjie Wang, Prof. Dr. Alim Abdurahman, Prof. Dr. Xiaomin Liu, Prof. Dr. Geyu Lu

State Key Laboratory of Integrated Optoelectronics, JLU Region, College of Electronic Science and Engineering, Jilin University, Qianjin Avenue 2699, 130012 Changchun, Jilin (P. R. China)

E-mail: alim@jlu.edu.cn; xiaominliu@jlu.edu.cn; lugy@jlu.edu.cn

[b] Dr. Li Shen

College of Chemical Engineering and Environmental Chemistry, Weifang University, Weifang, 261061, China

† These authors contributed equally to this work.

Contents

S1. Synthesis and Characterization of molecules

S2. Synthesis and Characterization of nanoparticles (NPs)

S3. In vitro and in vivo correlation tests

S4. Remaining Supplementary Figures and Tables

S4.1 EPR spectra of TT-CzPh and THT-CzPh powder

S4.2 Photophysical properties of TT-CzPh and THT-CzPh

S4.3 Theoretical calculations of radicals

S4.4 Photothermal performance of radicals

S4.5 The particle size of TTM-TTM NPs

S4.6 EPR spectra of NPs

S4.7 Normalized absorption and PL spectra of TT-CzPh NPs and TTM-TTM NPs

S4.8 Image of NPs taken by smartphone

S4.9 Photothermal performance of TTM-TTM NPs

S4.10 Photothermal capacity of TT-CzPh NPs

S4.11 Photothermal stability of TT-CzPh NPs

S4.12 Stability of TT-CzPh NPs over time

S4.13 In vitro cellular experiments with TT-CzPh NPs

S4.14 Fluorescence stability of TT-CzPh NPs

S4.15 In vivo experiments with TT-CzPh NPs

S5. Detailed data of TT-CzPh excited states calculated by TD-DFT

S1. Synthesis and Characterization of molecules

Main Materials

All chemical agents and solvents, unless otherwise stated, were purchased from commercial suppliers and used directly without further purification. Column chromatography was performed with silica gel (200-300 mesh).

Instruments

The ^{1}H nuclear magnetic resonance (NMR) spectra were recorded in deuterated dichloromethane (CD_2Cl_2) on a Bruker Avance-III 500 NMR spectrometer at ambient temperature. Matrix-assisted laser desorption/ionization time-of-flight (MALDI-TOF) mass spectra were recorded on a Bruker Autoflex speed TOF/TOF mass spectrometer with DCTB as a matrix. High-resolution MALDI-TOF mass spectra were measured with a Brucker Autoflex speed TOF spectrometer. High-performance liquid chromatography (HPLC) experiments were conducted on an Agilent 1260 Infinity II HPLC-MS equipped with an InfinityLab Poroshell 120. Electron paramagnetic resonance (EPR) spectra were recorded on a Bruker ELEXSYS-II E500 CW-EPR spectrometer. Ultraviolet-visible (UV-Vis) and photoluminescence (PL) spectra of the radicals were recorded on a Shimadzu UV-2550 spectrophotometer and a HITACHI F-4700 spectrophotometer. The PLQYs and the fluorescence lifetimes were measured with FLS980.

Synthesis and Characterization Data for the TT-CzPh

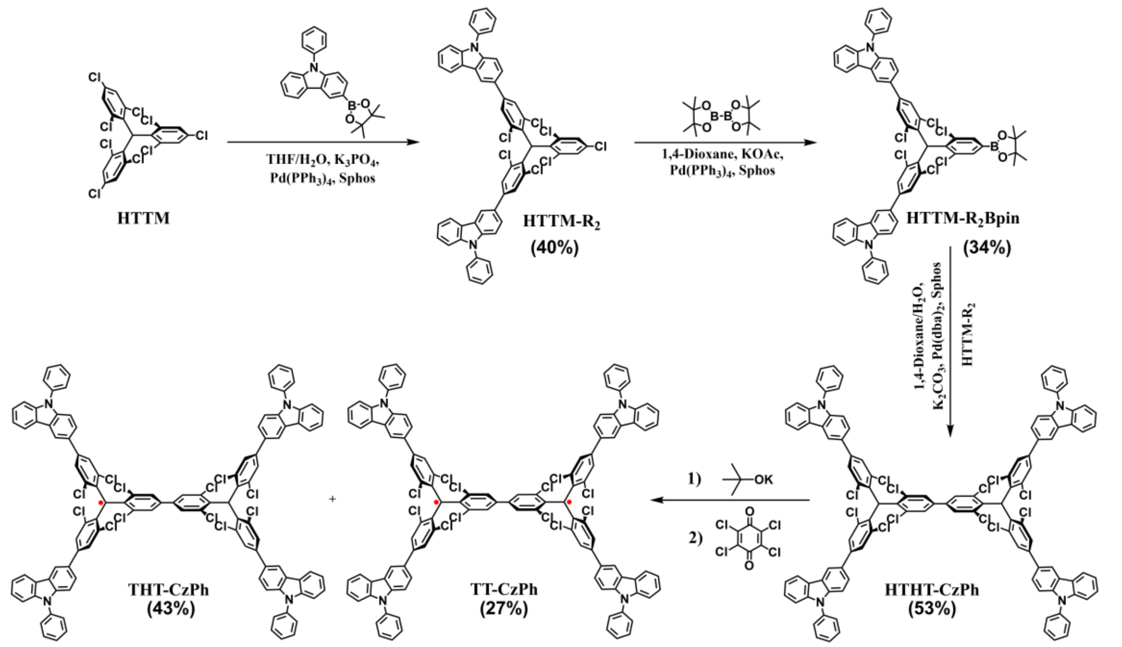

Scheme 1. Synthetic routes are toward **TT-CzPh**.

Synthesis of compound HTTM-R$_2$

HTTM was prepared according to our previous report[1-3]. Under argon atmosphere, HTTM (4.0 g, 7.2 mmol), 9-Phenyl-3-(4,4,5,5-tetramethyl-1,3,2-dioxaborolan-2-yl)-9H-carbazole (6.6 g, 18 mmol), Sphos (148.0 mg, 0.36 mmol), and Pd(PPh$_3$)$_4$ (416.0 mg, 0.36 mmol) were mixed in a double-neck flask containing THF (200 mL), and K_3PO_4 aqueous solution (50 mL, 2 M), the mixture was refluxed at 90 °C for 24 h. After the reaction mixture cooling to room temperature, the reaction mixture was extracted with dichloromethane. The organic extracts were dried over anhydrous $MgSO_4$. The crude product was further purified by silica gel column (dichloromethane : petroleum = 1: 6) to obtain **HTTM-R$_2$** as white powder (2.8 g, 40%). **^{1}H NMR** (500 MHz, CD_2Cl_2) δ 8.42 (s, 1H), 8.21 (d, J = 7.8 Hz, 1H), 7.80 (s, 1H), 7.69 (d, J = 8.7 Hz, 1H), 7.67 – 7.63 (m, 3H), 7.61 (d, J = 7.5 Hz, 2H), 7.51 (dd, J = 16.8, 8.0 Hz, 2H), 7.45 (d, J = 6.0 Hz, 2H), 7.33 (t, J = 6.5 Hz, 2H), 6.93 (s, 1H). **^{13}C NMR** (125 MHz, CD_2Cl_2) δ 143.44, 142.29, 141.77, 139.02, 138.63, 138.45, 138.14, 137.89, 137.81, 135.93, 134.17, 133.96, 130.78, 130.41, 129.17, 128.51, 127.79, 127.51, 127.25, 125.66, 124.76, 124.02, 121.15, 119.47, 111.11, 110.83, 51.11, 30.48. **MALDI-TOF-MS** (m/z): Calculated for $C_{55}H_{31}Cl_7N_2$: 968.02, Found [M$^+$]: 967.95.

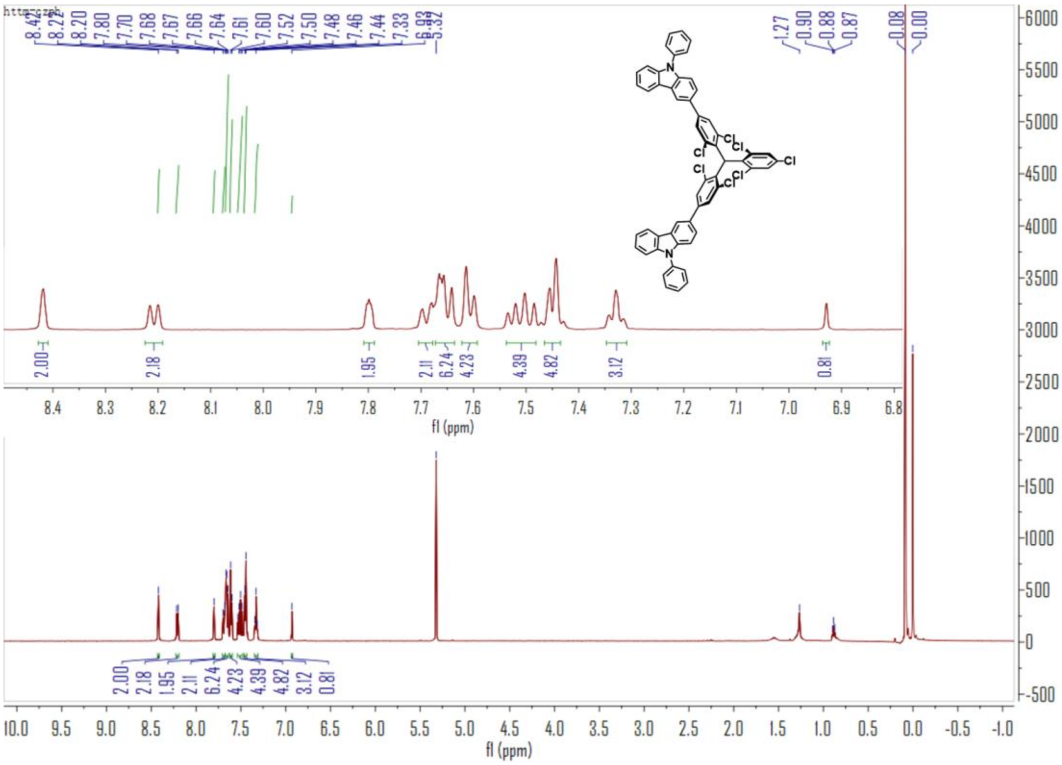

Fig. S1 ^{1}H NMR spectrum (500 MHz) of compound **HTTM-R$_2$** in CD_2Cl_2 at 298 K. The solvent peak residuals corresponding to n-hexane are at 0.9 ppm and 1.27 ppm.

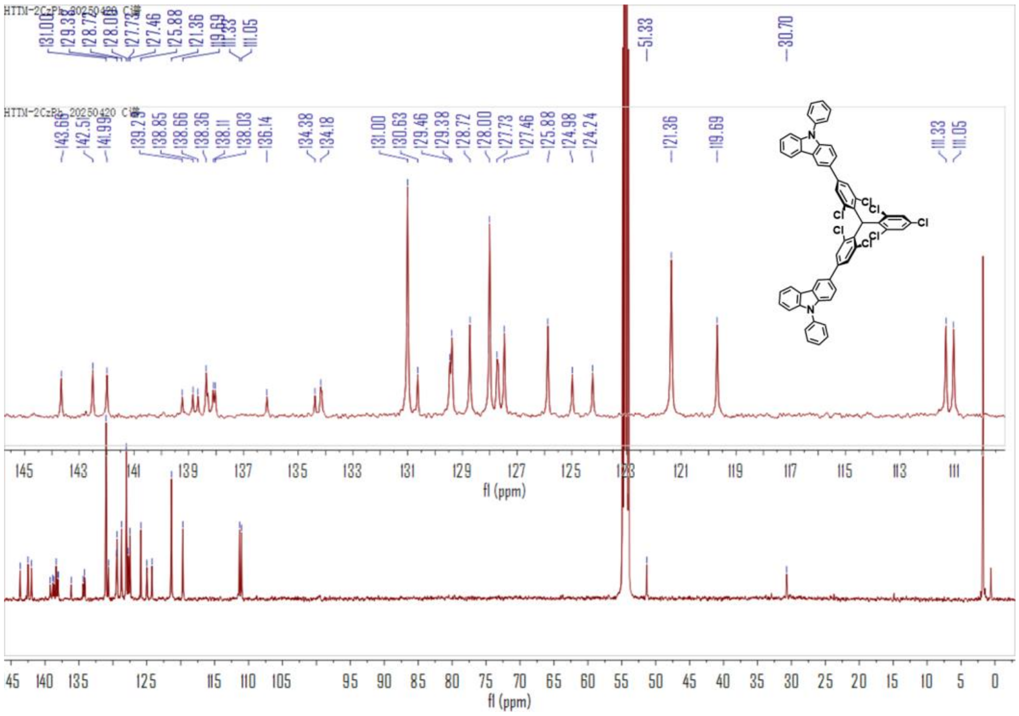

Fig. S2 ^{13}C NMR spectrum (125 MHz) of compound **HTTM-R$_2$** in CD_2Cl_2 at 298 K.

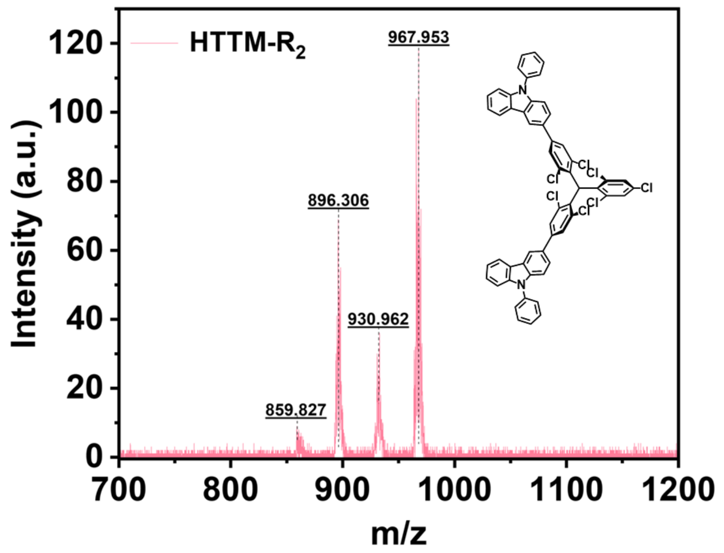

Fig. S3 MALDI-TOF MS spectrum of compound **HTTM-R2**.

Synthesis of compound HTTM-R2Bpin

Under argon atmosphere, **HTTM-R2** (1.4 g, 1.5 mmol), 4,4,4',4',5,5,5',5'-octamethyl-2,2'-bi(1,3,2-dioxaborolane) (571.3 mg, 2.25 mmol), Sphos (61.6 mg, 0.15 mmol), $Pd(PPh_3)_4$ (51.8 mg, 0.09 mmol) and KOAc (265.0 mg, 2.7 mmol) were mixed in a double-neck flask containing 1,4-Dioxane (35.0 mL), the mixture was refluxed at 80 °C for 24 h. After the reaction mixture cooling to room temperature, the reaction mixture was extracted with dichloromethane. The organic extracts were dried over anhydrous $MgSO_4$. The crude product was further purified by silica gel column (ethyl acetate: petroleum = 1: 8) to obtain **HTTM-R2Bpin** as white powder (540.4 mg, 34%). **^{1}H NMR** (500 MHz, CD_2Cl_2) δ 8.46 (s, 2H), 8.25 (d, *J* = 7.7 Hz, 2H), 7.83 (dd, *J* = 8.8, 1.6 Hz, 2H), 7.80 (s, 1H), 7.73 (d, *J* = 8.7 Hz, 2H), 7.71 – 7.67 (m, 7H), 7.65 (d, *J* = 7.5 Hz, 4H), 7.55 (dd, *J* = 16.6, 8.0 Hz, 4H), 7.49 (d, *J* = 6.0 Hz, 4H), 7.37 (t, *J* = 6.6 Hz, 2H), 7.02 (s, 1H), 1.39 (s, 12H). **^{13}C NMR** (125 MHz, CD_2Cl_2) δ 143.07, 142.06, 141.53, 139.34, 138.46, 138.35, 137.95, 137.73, 137.63, 137.17, 136.55, 134.67, 134.15, 130.56, 130.29, 128.96, 128.90, 128.27, 127.57, 127.21, 127.00, 125.46, 124.53, 123.82, 120.94, 120.90, 119.25, 110.87, 110.60, 85.08, 51.46, 30.27, 25.27, 25.21. **MALDI-TOF-MS** (m/z): Calculated for $C_{61}H_{43}BCl_6N_2O_2$: 1059.54, Found [M^+]: 1059.73.

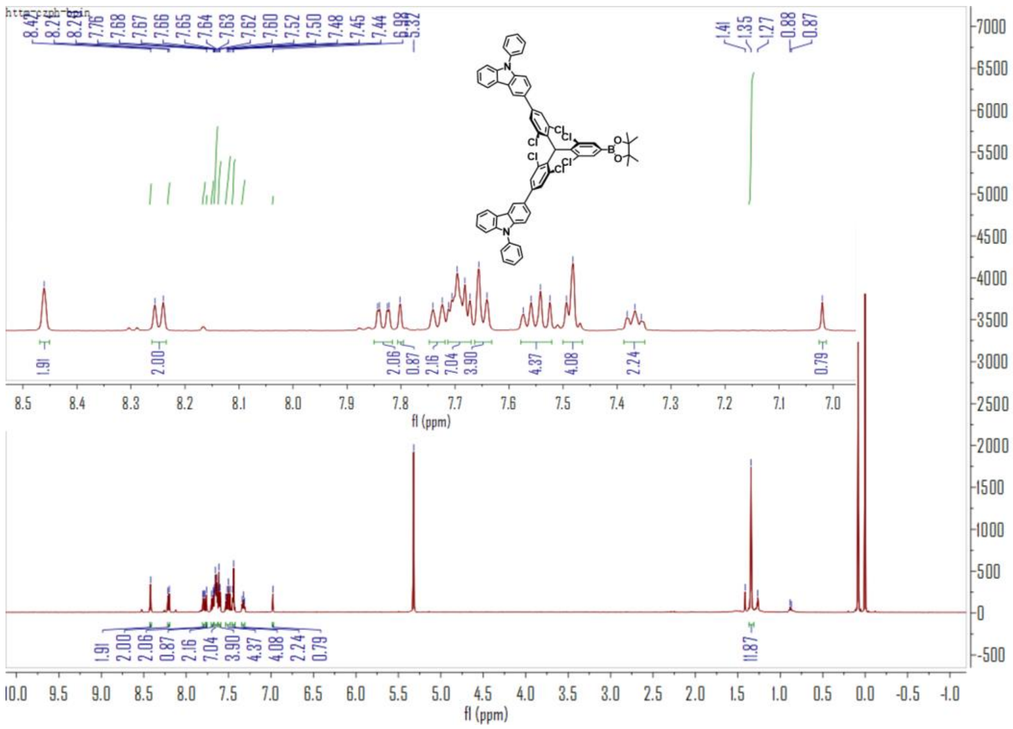

Fig. S4 ^{1}H NMR spectrum (500 MHz) of compound **HTTM-R₂Bpin** in CD_2Cl_2 at 298 K. The solvent peak residuals corresponding to n-hexane are at 0.9 ppm and 1.27 ppm, while the solvent peak residual corresponding to cyclohexane is at 1.41 ppm.

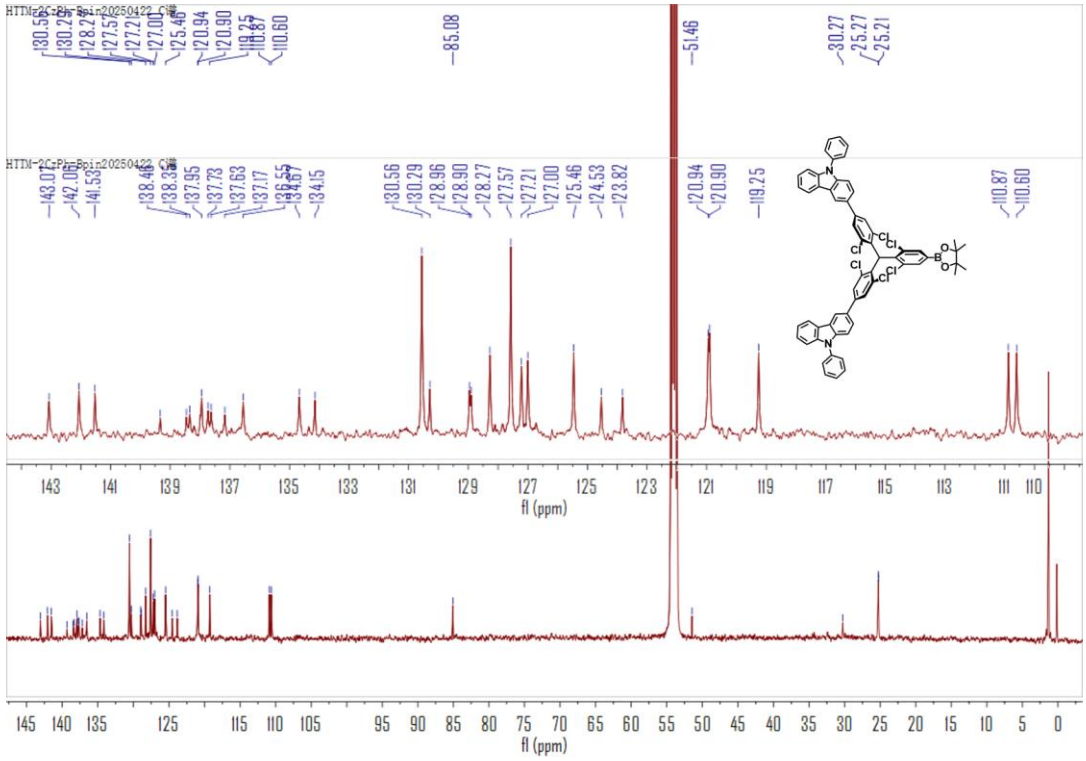

Fig. S5 ^{13}C NMR spectrum (125 MHz) of compound **HTTM-R$_2$Bpin** in CD_2Cl_2 at 298 K.

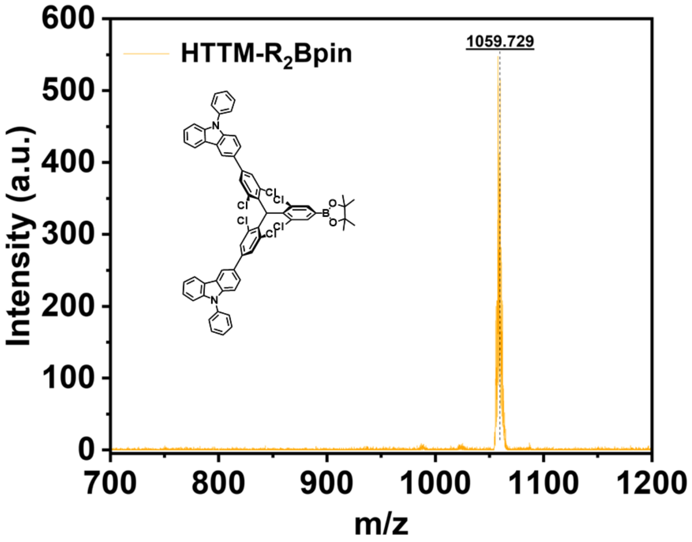

Fig. S6 MALDI-TOF MS spectrum of compound **HTTM-R$_2$Bpin**.

Synthesis of compound HTHT-CzPh

Under argon atmosphere, **HTTM-R$_2$** (484.0 mg, 0.5 mmol), **HTTM-R$_2$Bpin** (530.0 mg, 0.5 mmol), Sphos (12.3 mg, 0.03 mmol), and Pd(dba)$_2$ (11.5 mg, 0.02 mmol) were mixed in a double-neck flask containing 1,4-Dioxane (20.0 mL), and K_2CO_3 aqueous solution (5.0 mL, 2 M), the mixture was refluxed at 80 °C for 24 h. After the reaction mixture cooling to room temperature, the reaction mixture was extracted with dichloromethane. The organic extracts were dried over anhydrous $MgSO_4$. The crude product was further purified by silica gel column (dichloromethane : petroleum = 1 : 2.5) to obtain **HTHT-CzPh** as white powder (494.3 mg, 53%). **^{1}H NMR** (500 MHz, CD_2Cl_2) δ 8.43 (s, 4H), 8.21 (d, J = 7.8 Hz, 4H), 7.82 (s, 4H), 7.72 (d, J = 5.3 Hz, 4H), 7.68 (t, J = 5.4 Hz, 7H), 7.65 (d, J = 7.5 Hz, 7H), 7.63 – 7.58 (m, 10H), 7.51 (dd, J = 14.2, 7.9 Hz, 8H), 7.47 – 7.42 (m, 8H), 7.33 (t, J = 6.7 Hz, 4H), 7.02 (s, 2H). **^{13}C NMR** (125 MHz, CD_2Cl_2) δ 143.18, 142.07, 141.55, 138.95, 138.88, 138.46, 138.35, 138.08, 137.94, 137.73, 136.71, 134.00, 130.57, 130.26, 128.96, 128.28, 127.58, 127.28, 127.02, 125.47, 124.55, 123.82, 120.92, 119.27, 110.90, 110.61, 51.16, 30.27. **MALDI-TOF-MS** (m/z): Calculated for $C_{110}H_{62}Cl_{12}N_4$: 1865.13, Found [M^+]: 1865.61. **HR-MALDI-TOF-MS** is reported in Fig. S10.

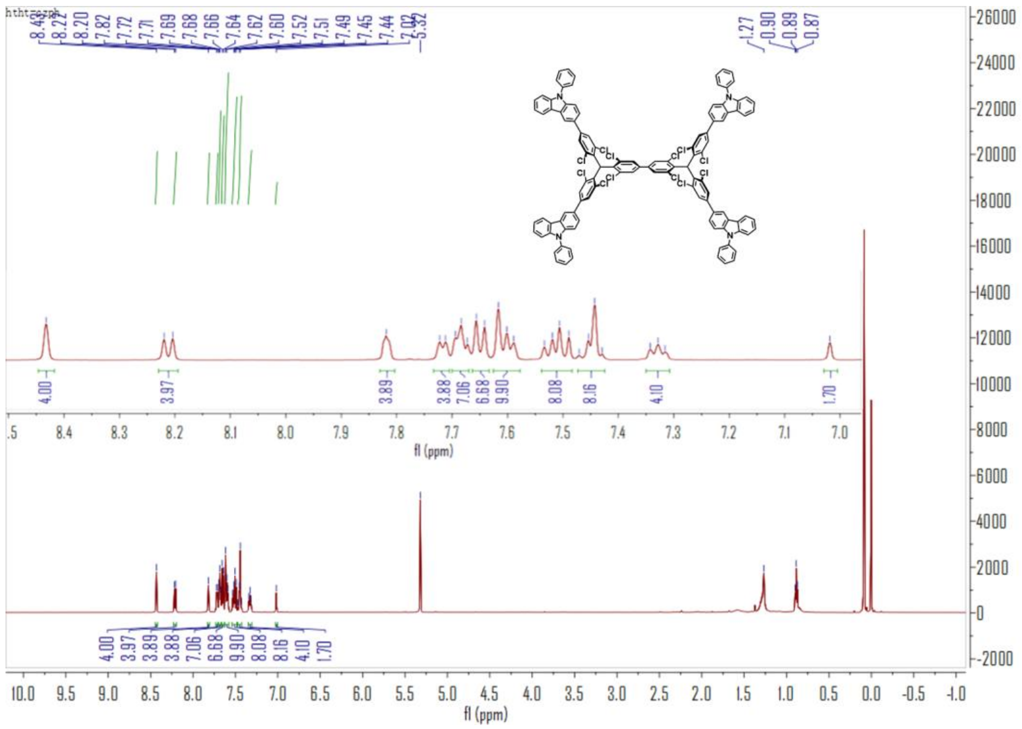

Fig. S7 ^{1}H NMR spectrum (500 MHz) of compound **HTHT-CzPh** in CD_2Cl_2 at 298 K. The solvent peak residuals corresponding to n-hexane are at 0.9 ppm and 1.27 ppm.

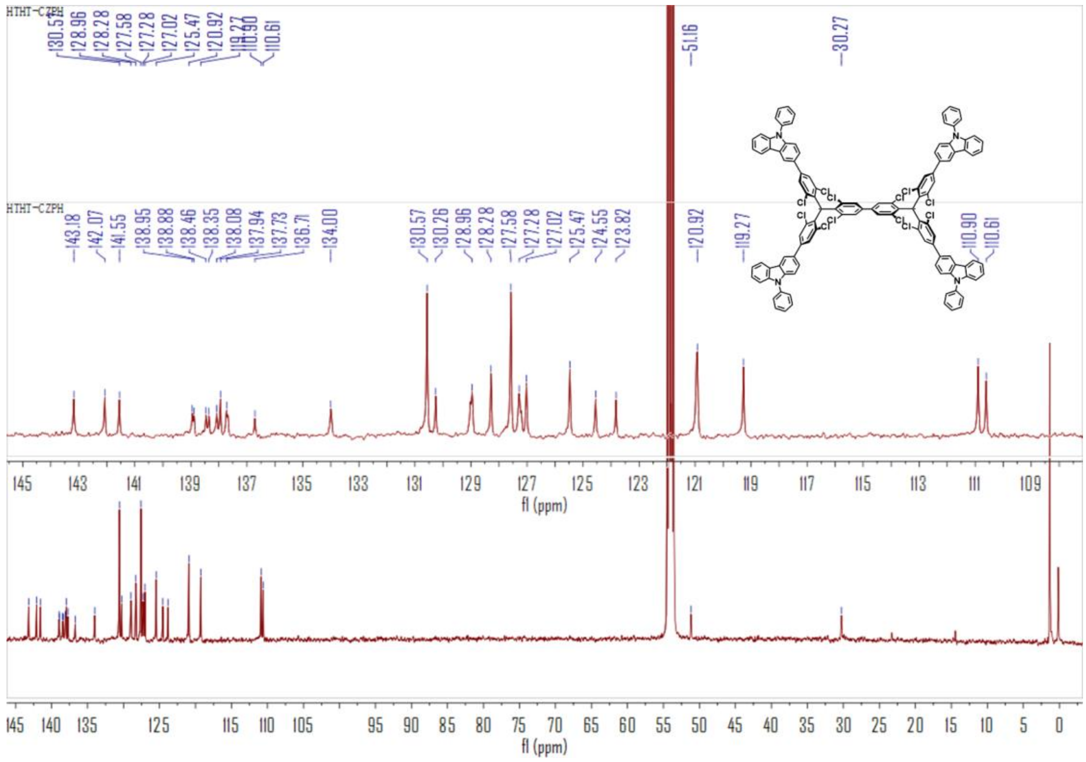

Fig. S8 ^{13}C NMR spectrum (125 MHz) of compound **HTHT-CzPh** in CD_2Cl_2 at 298 K.

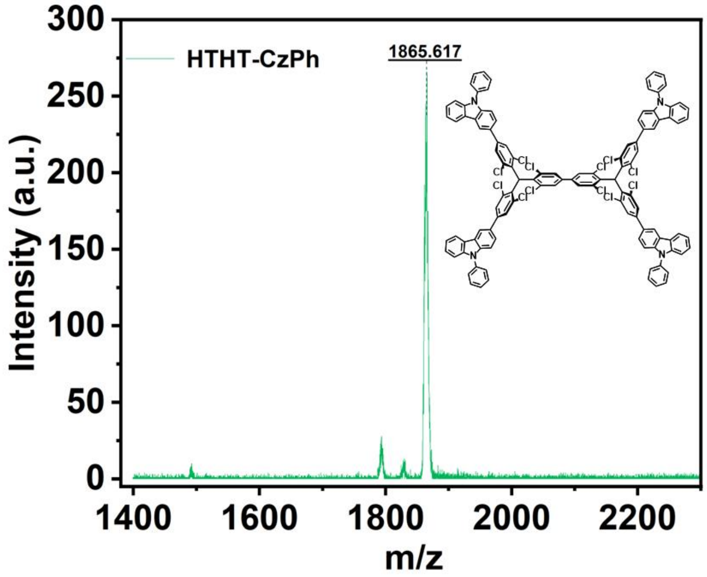

Fig. S9 MALDI-TOF MS spectrum of compound **HTHT-CzPh**.

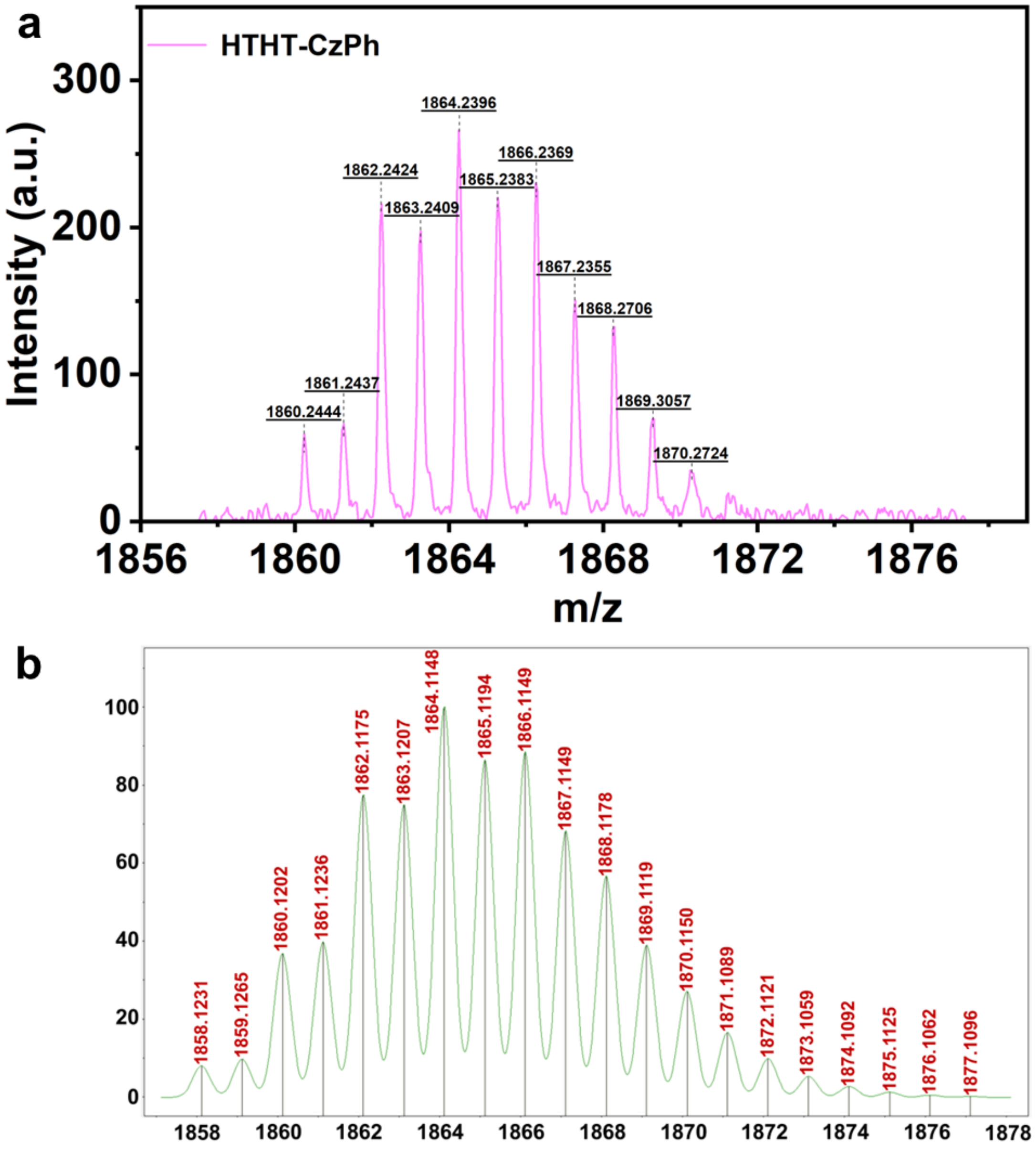

Fig. S10 HR-MALDI-TOF-MS with the corresponding experimental **a** and simulated **b** isotopic distributions of **HTHT-CzPh**.

Synthesis of TT-CzPh and THT-CzPh

Under argon atmosphere and in the dark, the KOtBu (1.5 g, 13.3 mmol) was added to the solution of **HTHT-CzPh** (0.3 g, 0.16 mmol) in dry THF. The solution was stirred for 15 h at room temperature, and then the 2,3,5,6-tetrachloro-p-benzoquinone (3.3 g, 13.3 mmol) was added. The solution was stirred for another 6 h. After the reaction finished, the solvent was removed under vacuum and further purified by silica gel column chromatography (dichloromethane: petroleum = 1: 3) to obtain **THT-CzPh** as green solid (128.2 mg, 43%) and **TT-CzPh** as dark bluish-green solid (80.5 mg, 27%).

THT-CzPh: MALDI-TOF-MS (m/z): Calculated for $C_{110}H_{61}Cl_{12}N_4$: 1864.13, Found [M^+]: 1864.77. **HR-MALDI-TOF-MS** is reported in Fig. S13.

TT-CzPh: MALDI-TOF-MS (m/z): Calculated for $C_{110}H_{60}Cl_{12}N_4$: 1863.12, Found [M^+]: 1863.36. **HR-MALDI-TOF-MS** is reported in Fig. S15. **Elem. Anal.** Calcd for $C_{110}H_{60}Cl_{12}N_4$: C 70.91, H 3.25, N 3.01. Found, C 71.04, H 3.42, N 2.75.

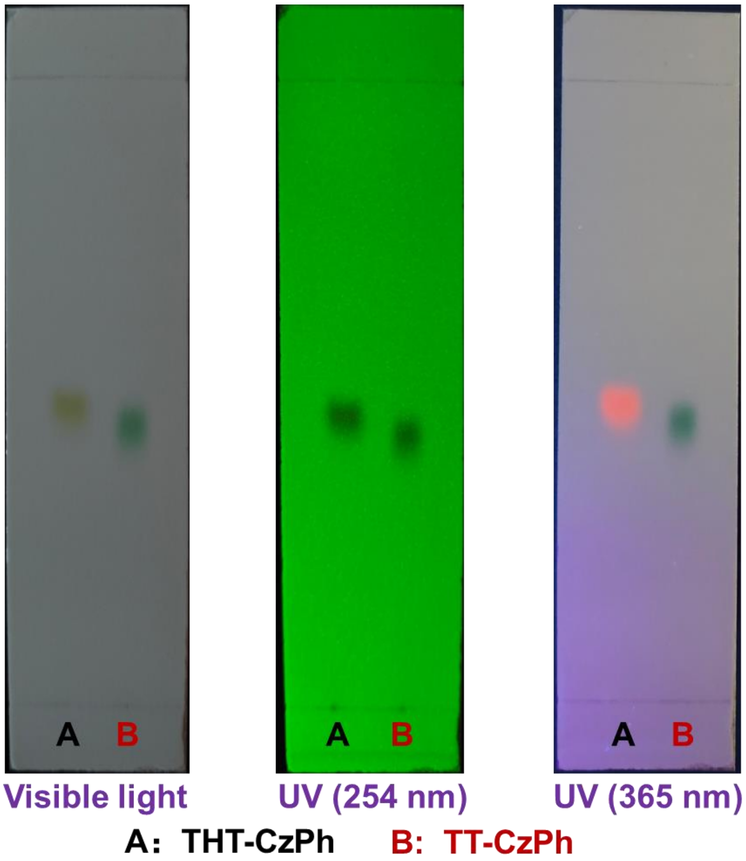

Fig. S11 Thin Layer Chromatography (TLC) of **THT-CzPh** and **TT-CzPh**.

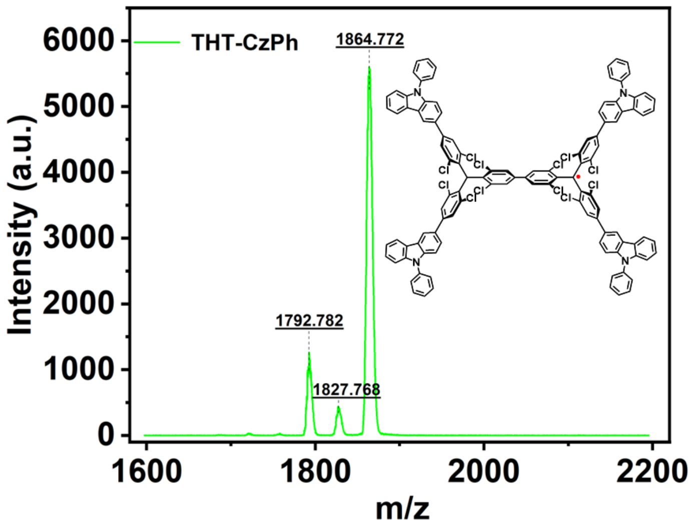

Fig. S12 MALDI-TOF MS spectrum of compound **THT-CzPh**.

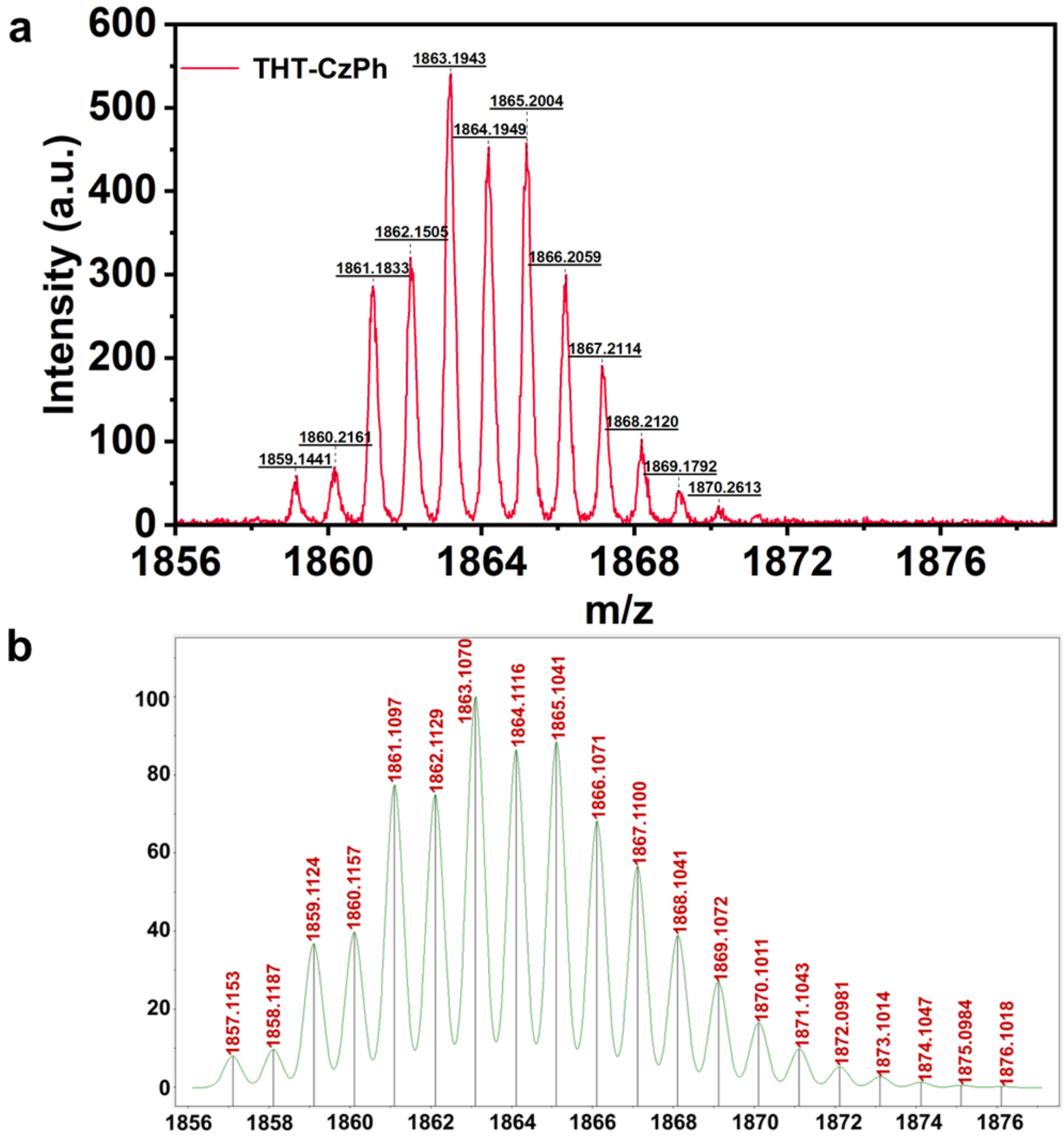

Fig. S13 HR-MALDI-TOF-MS with the corresponding experimental **a** and simulated **b** isotopic distributions of **THT-CzPh**.

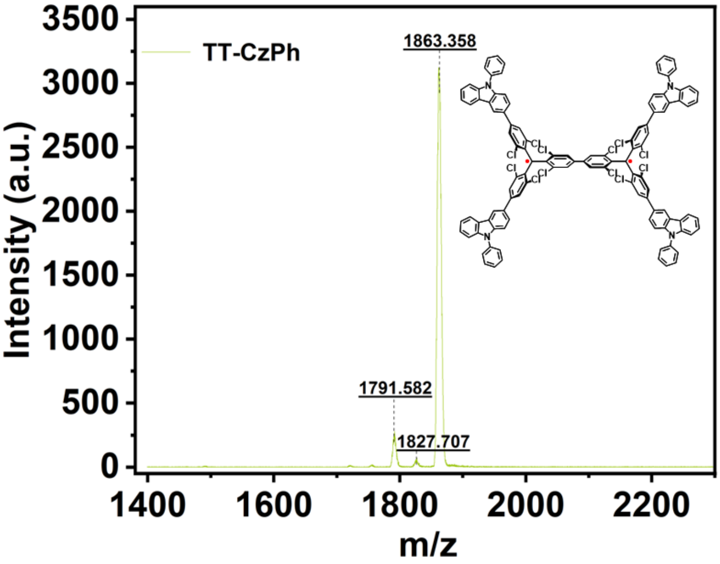

Fig. S14 MALDI-TOF MS spectrum of compound **TT-CzPh**.

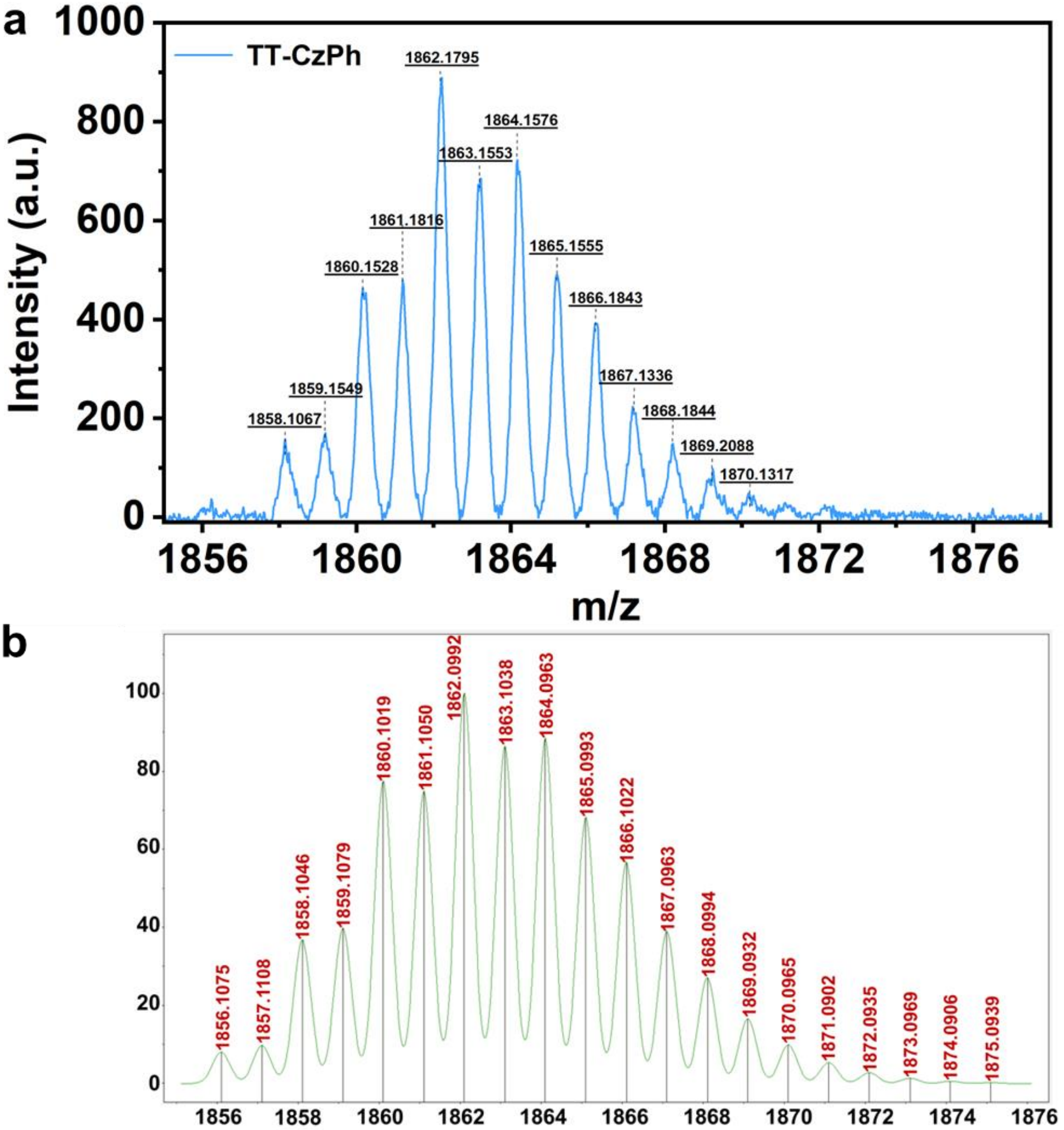

Fig. S15 HR-MALDI-TOF-MS with the corresponding experimental **a** and simulated **b** isotopic distributions of **TT-CzPh**.

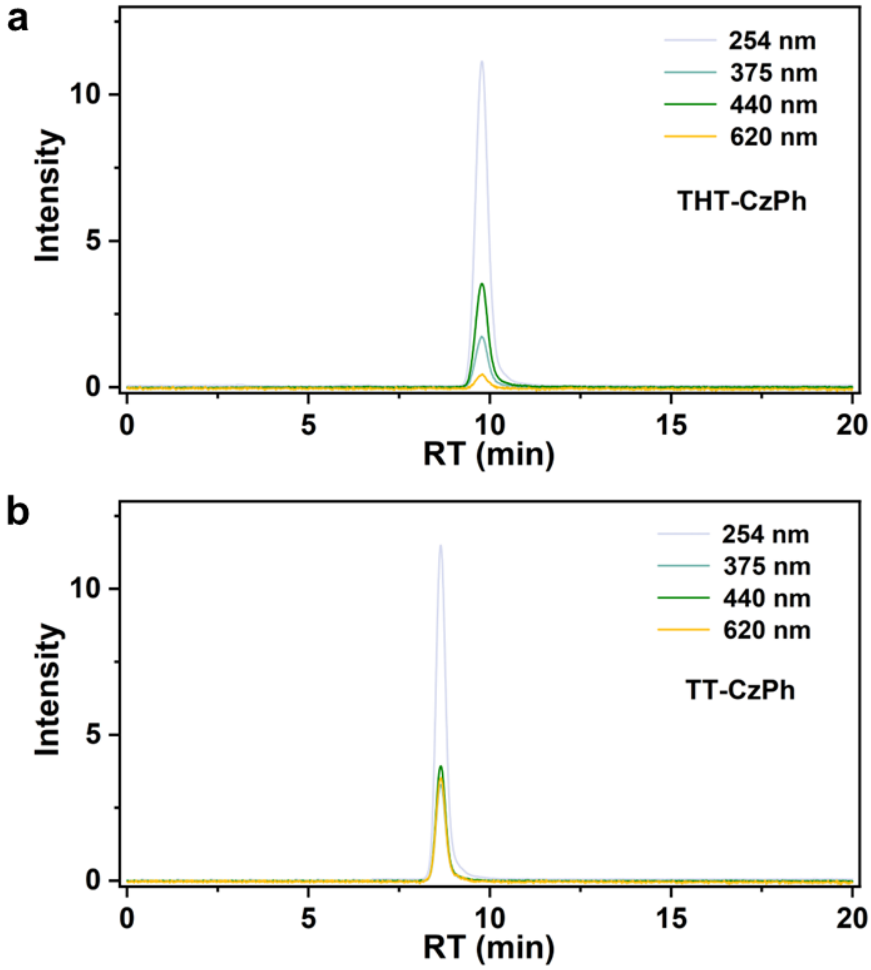

Fig. S16 High performance liquid chromatography (HPLC) of **a THT-CzPh** and **b TT-CzPh**. Mobile phase: Acetonitrile/isopropanol = 6/4, v/v; Flow rate: 0.5 mL/min; Injection volume: 10 μL.

Variable-temperature (VT) EPR measurements

The VT-EPR data was fitted by modified Bleaney-Bowers equation:

$$IT = \frac{C}{k_B[3 + \exp\left(-\frac{2J}{k_B T}\right)]}$$

Where C is a constant and -2J is correlated to the excitation energy from the ground state to the first excited state.

S2. Synthesis and Characterization of nanoparticles (NPs)

Main Materials

1, 2-Distearoyl-sn-glycero-3-phosphoethanolamine-N-[methoxy (polyethylene glycol) - 2000] (DSPE-mPEG$_{2000}$) was purchased from Aladdin. Calcein AM/PI Dual Staining Kit, mitochondrial membrane potential assay kit (JC-1) by Beijing Solarbio Sciences & Technology Co., Ltd. DMEM medium, Fetal bovine serum (FBS), Trypsin, Penicillin and Streptomycin were purchased from Dalian Meilun Biotechnology Co., Ltd.

Instruments

The size distribution and zeta potential were analyzed by dynamic light scattering (DLS) using a Zetasizer-Nano ZSZEN3600 apparatus (Malvern Instruments, UK). The nanoparticles (NPs) of particle size and morphology were determined using a JEM-2100 transmission electron microscope (TEM). The cell confocal images were obtained using a two-photon Nikon A1RMP microscope. Photothermal images and temperature variation were captured by a FLIR E8-XT camera (FLIR Systems). In vivo imaging and organ imaging were performed using an in vivo imaging chamber (SRAR-BIOGENII, JXXQ) provided by Changchun Jixian Xingqun Technology Co., Ltd.

Preparation of TT-CzPh NPs

Based on the amphiphilic self-assembly characteristics of DSPE-mPEG$_{2000}$, TT-CzPh is encapsulated in the hydrophobic core. The specific method is as follows: TT-CzPh (0.5 mg mL^{-1}, 500 μL) in THF and DSPE-mPEG$_{2000}$ (5.0 mg mL^{-1}, 500 μL) in THF were sonicated for 10 min, then mixed homogeneously and sonicated for another 10 min. Under vigorous stirring, 1 mL of the above mixed solution was added dropwise to 9 mL of ultrapure water. After stirring for 48 h at room temperature, the solution was filtered through a 0.22 μm microporous membrane. The solution was then dialyzed using a dialysis bag (MWCO 3.5 kDa) for 48 h. The water was changed every 4 h during the first 24 h of dialysis. At the end of dialysis, it was concentrated using ultrafiltration centrifuge tubes (Pall, MWCO 3 kDa). Finally, the concentrated TT-CzPh NPs dispersion was stored at 4 °C in a dark place.

Preparation of TTM-TTM NPs

The synthesis steps were consistent with those of TT-CzPh NPs.

Photothermal performance characterization

A 655 nm light was used to irradiate TTM-TTM, TT-CzPh, TTM-TTM NPs and TT-CzPh NPs (1.2 mL). Temperature changes of TTM-TTM and TT-CzPh dissolved in DMSO (1.0 W cm^{-2}, 25 μg mL^{-1}) were detected using a FLIR E8-XT camera. The stability of TT-CzPh (25 μg mL^{-1}) was verified by observing 5 cycles of laser irradiation (1.0 W cm^{-2}) and natural cooling to room temperature.

A FLIR E8-XT thermal imaging camera was used to examine the temperature changes of two NPs with different concentrations (1.0 W cm^{-2}, 0, 10, 20, 30, 40 and 50 μg mL^{-1}) and different laser power intensities (50 μg mL^{-1}, 0.25, 0.5, 0.75 and 1.0 W cm^{-2}). The stability of TT-CzPh NPs (50 μg mL^{-1}) was verified by observing 5 cycles of laser irradiation (1.0 W cm^{-2}) and natural cooling to room temperature.

The temperature profile of TTM-TTM, TT-CzPh, TTM-TTM NPs and TT-CzPh NPs are obtained under 655 nm laser irradiation (1 W cm^{-2}). The cooling curve is obtained by: turning off the laser light source when the sample temperature rises to an appropriate level (with a slow heating rate), then recording the sample temperature every 20 seconds until it completely returns to room temperature. During each test, the positions of the laser, sample stage and thermal camera are all fixed by tripods and positioning marks to ensure repeatability. The thermal camera was placed approximately 20 cm away from the sample cuvette. The sample solution was contained in a custom cylindrical quartz cuvette (13 mm in diameter, 15 mm in height, 1 mm wall thickness, 1.3 mL in volume). During testing, the laser was vertically irradiated onto the surface of the sample solution and penetrates the cuvette containing 1.2 mL of the sample solution.

The photothermal conversion efficiency was determined according to Equation (1), and the other parameters in Equation (1) were calculated from Equations (2), (3) and (4)[4].

$$\eta = \frac{hS(T_{Max}-T_{Surr})-Q_{Dis}}{I(1-10^{-A_{655}})} \qquad \ldots\ldots (1)$$

$$\tau_s = \frac{\sum_i m_i C_{p,i}}{hS} \qquad \ldots\ldots (2)$$

$$t = -\tau_s \ln(\theta) \qquad \ldots\ldots (3)$$

$$\theta = \frac{T - T_{Surr}}{T_{Max} - T_{Surr}} \qquad \ldots\ldots (4)$$

In Equation (1), η is the photothermal conversion efficiency (PCE), h is the heat transfer coefficient, and S is the surface area of the container. Q_{Dis} represents the heat dissipated from the laser mediated by the solvent and container. I is the laser power, and A_{655} is the absorbance of the sample at 655 nm. In Equation (2), m is the mass of the solution containing the photoactive material, and C is the specific heat capacity of the solution (C_{DMSO} = 2.9 J g^{-1}, C_{water} = 4.2 J g^{-1}). In Equation (3), τ_s is the associated time constant. In Equation (4), θ is a dimensionless parameter known as the driving force temperature. T_{max} and T_{Surr} are the maximum steady-state temperature and the environmental temperature, respectively.

S3. In vitro and in vivo correlation tests

Cell Culture

Mouse breast cancer cells (4T1) and human hepatocellular carcinoma (HepG2) were purchased from the cell bank of the Chinese Academy of Sciences (Shanghai, China). Mouse breast cancer cells (4T1) and human hepatocellular carcinoma (HepG2) were cultured in Dulbecco's modified Eagle's medium (DMEM, high glucose, pyruvate) containing 10% fetal bovine serum (FBS) and 1% Antibiotic-Antimycotic (AA) at 37°C in a humidified 5% (vol/vol) CO_2 incubator.

Cellular Uptake

We assessed cellular uptake via electron paramagnetic resonance (EPR) spectroscopy, leveraging the unpaired electrons of diradicals. The procedure was as follows:

4T1 or HepG2 cells were plated in culture dishes and cultured for 24 h, then incubated with TT-CzPh NPs (0 or 100 μg mL^{-1} in DMEM+) for 4 h. Next, the cells were rinsed three times with PBS to remove suspended, non-internalized NPs. Trypsin was used to detach the cells and form a suspension, which was concentrated using ultrafiltration centrifuge tubes. Finally, EPR signals from the concentrated suspension were detected

via EPR spectroscopy to evaluate cellular uptake.

Cell Viability

The effect of TT-CzPh NPs on cell viability was analyzed using 3-(4,5-dimethylthiazol-2-yl)-2,5-diphenyltetrazolium bromide (MTT). Cells were seeded into a flat-bottomed 48-well plate (2×10^4 cells/well), incubated in DMEM containing 10% FBS and 1% AA at 37°C in a 5% (vol/vol) CO_2 incubator for 24 h. After rinsing with DMEM, fresh DMEM medium containing TT-CzPh NPs with varied concentrations (0, 10, 20, 30, 40 and 50 μg mL^{-1}) was added. The cells were then incubated for an additional 4 h. Sequentially, the cells were exposed to 655 nm laser irradiation (1.0 W cm^{-2}) for 5 min. Another set of cells incubated with TT-CzPh NPs without laser irradiation was assessed for cytotoxicity under dark conditions. After further incubation for 4 h, the media was removed, and the cells were washed with PBS three times. Then, MTT reagent (20 μL) was added to each well, and the plates were incubated for another 4 h in a CO_2 incubator. Excess MTT tetrazolium solution was then removed. After the formazan crystals were solubilized in DMSO (300 μL/well) for 15 min at room temperature, the absorbance of each well was measured by a microplate reader (Bio-Tek Instruments, Inc) with an excitation at 490 nm. The results were expressed as the percentage of viable cells after different treatments relative to that of the control cells without any treatment. The relative cell viability was calculated according to the following formula:

$$\text{Cell viability (\%)} = (OD_{sample} - OD_{background}) / (OD_{control} - OD_{background}) \times 100\%$$

Live/dead cell staining

The live/dead co-staining was carried out using Calcein-AM/PI and Annexin V-FITC/PI Live/Dead Viability/Cytotoxicity Assay Kits for CLSM imaging and flow cytometry analysis. First, 4T1 cells were inoculated into confocal glass substrate culture dishes and cultured in an incubator for 24 h. Then, the medium was replaced with fresh DMEM medium containing TT-CzPh NPs (50 μg mL^{-1}) and cultured for 4 hours. The cells in the PBS + L group and NPs + L group were irradiated with 655 nm laser (1.0 W cm^{-2}, 5 min). After laser treatment, the cells were further incubated for 4 h. Finally, the cells were rinsed with PBS and stained with 1 μM Calcein-AM and 1 μM PI for 20

min. The residual dye was removed by washing with PBS three times. Confocal fluorescence microscopy was used to observe the green fluorescence of Calcein-AM, indicating live cells, and the red fluorescence of PI, indicating dead cells.

The cells apoptosis and necrosis analysis were determined by Annexin V-FITC Apoptosis Detection Kit and flow cytometry.

In vitro mitochondrial function assay

The mitochondrial membrane potential (MMP) was detected by the enhanced mitochondrial membrane potential assay kit with JC-1 fluorescent probe. The cell culture procedure was the same as that used for live/dead cell staining. After irradiation with 655 nm light or kept in the dark, the cells were incubated in the incubator for 4 h. After staining with JC-1 for 20 min, the cells were washed with PBS three times, and finally, confocal microscope imaging was performed. The green fluorescence of the JC-1 monomer (JC-M) was excited at 426-466 nm and collected at 511-551 nm. The red fluorescence of JC-1 aggregates (JC-A) was excited at 500-580 nm and collected at 594-900 nm.

Animals and tumor model

BALB/c mice (5-6 weeks old, female) were purchased from Liaoning Chang sheng Biotechnology Co. All animal experiments were performed in compliance with the guidelines of the Care and Use of Laboratory Animals and approved by the Experimental Animal Ethics Committee, College of Basic Medicine, Jilin University (approval number: SYXK2023-0010). The mice were subcutaneously administrated with 100 μL of 4T1 cell suspension (1×10^6) into the right forelimb of the mice. When the tumor volume reached about 70 mm^3, these tumor-xenograft mice were used to follow experiments.

Tumor volumes were measured every two days using a vernier caliper and calculated using the following formula:

Volume = ((tumor length) × (tumor width)^2)/2

In vivo fluorescence imaging

4T1 tumor-bearing mice were injected with TT-CzPh NPs (500 μg mL^{-1}, 200 μL).

Fluorescence images of the mice were then acquired using an in vivo imager with a 700 nm long-pass filter at various time points after injection (0, 1, 6, 9, 12, 24, 36, and 48 h). Subsequently, the mice were euthanized, and the main organs (heart, liver, spleen, lung and kidney) and tumor were removed. Then, the fluorescence intensities of isolated organs and tumors were measured using a near-infrared fluorescence imaging system.

In vivo photothermal cancer therapy

The 4T1 tumor-bearing mice were randomly divided into 4 groups (n = 6 for each group): “PBS - L”, “PBS + L”, “TT-CzPh NPs - L” and “TT-CzPh NPs + L”. For the 'PBS - L' and 'TT-CzPh NPs - L' groups, 200 μL of PBS or TT-CzPh NPs (500 μg mL^{-1}, determined by the amount of TT-CzPh) were injected into 4T1 tumor-bearing mice, respectively, without subsequent laser irradiation. The mice in the “PBS + L” and “TT-CzPh NPs + L” groups underwent laser irradiation with a 655 nm laser (1.0 W cm^{-2}) for 5 min at 9 h postinjection of 200 μL of PBS or TT-CzPh NPs (500 μg mL^{-1}, determined by the amount of TT-CzPh), respectively. The tumor volumes and body weights were measured every two days for 14 days. In addition, infrared thermal images of the mice were acquired using an FLIR E8-XT camera during irradiation with a 655 nm laser (1.0 W cm^{-2}) for 5 min at 9 h after administration of the TT-CzPh NPs. Mice injected with PBS under the same irradiation conditions were used as controls. The tumor size was measured by a vernier caliper, and the tumor volume (V) was estimated using the formula: Volume = ((tumor length) × (tumor width)^2)/2. Relative tumor volume was calculated as RTV = V/V_0 (V_0 was the initial tumor volume).

Hemolysis assay

Whole blood (0.5 mL) was obtained from BALB/c mice by enucleation of the eyes. After standing at room temperature for 3 h, the blood was centrifuged at 3000 rpm for 20 min at 4°C to harvest red blood cells (RBCs). The RBCs were washed three times with phosphate-buffered saline (PBS) and diluted to 10 mL with PBS. Subsequently, 0.2 mL of the red blood cell (RBC) suspension was added to 0.8 mL of TT-CzPh NPs at different concentrations (10, 20, 30, 40, 50, and 100 μg mL^{-1}, diluted with PBS (pH

7.4)) and mixed, with three replicates set for each group. The samples were incubated at room temperature for 1 h. All samples were then centrifuged at 3000 rpm for 5 min at 4°C, and the absorbance of the supernatant at 541 nm was measured to determine hemoglobin content. A mixture of 0.2 mL RBC suspension and 0.8 mL PBS was used as the negative control (0% hemolysis), while a mixture of 0.2 mL RBC suspension and 0.8 mL distilled water served as the positive control (100% hemolysis). A typical hemolysis percentage can be calculated as follows:

$$\text{Hemolysis (\%)} = \frac{A_{sample} - A_{0\%}}{A_{100\%} - A_{0\%}} \times 100\%$$

Histological Examination

After 14 days of treatment, the mice in the various groups (“PBS -L”, “PBS + L”, “TT-CzPh NPs - L” and “TT-CzPh NPs + L”) were sacrificed for further histological analysis of tumor tissues and normal organs (heart, liver, spleen, lung, and kidneys). These slices were fixed in PBS containing 4% paraformaldehyde, routinely processed into paraffin, sectioned at 4 μm. Normal organ tissues were stained with hematoxylin and eosin (H&E), while tumor tissues were stained with H&E and terminal deoxynucleotidyl transferase (TdT)-mediated dUTP nick-end labelling (TUNEL), followed by orthostatic microscope (model Ci-L) observation.

Statistical Analysis

Data from the cell viability test are presented as the mean ± standard deviation (s.d.) with n = 3. Statistical significance between the non-light and light groups was determined using t-tests, and P values were calculated using GraphPad Prism software (version 9). A p value < 0.05 was considered statistically significant, and data in the graphs are marked with *P < 0.05, **P < 0.01, ***P < 0.001, and ****P < 0.0001.

For the in vivo antitumor effect, the reported tumor volume after photothermal treatment is presented as the mean ± standard deviation (s.d.), n = 6. One-way analysis of variance (ANOVA) was performed for statistical analysis among multiple groups. P values were calculated using GraphPad Prism Software (version 9). The data in the figures were marked by *P < 0.05, **P < 0.01, ***P < 0.001, ****P < 0.0001. No animals were excluded from the analysis. No data were excluded from the analyses.

S4. Remaining Supplementary Figures and Tables

S4.1 EPR spectra of TT-CzPh and THT-CzPh powder

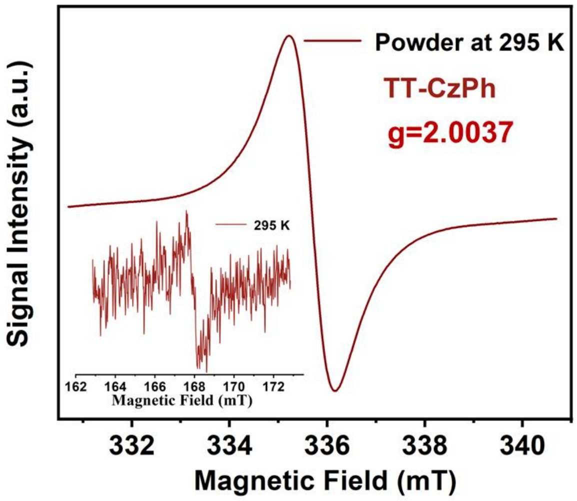

Fig. S17 EPR spectra of **TT-CzPh** powder at 295 K, the inset shows the $\Delta m_s = \pm 2$ resonance.

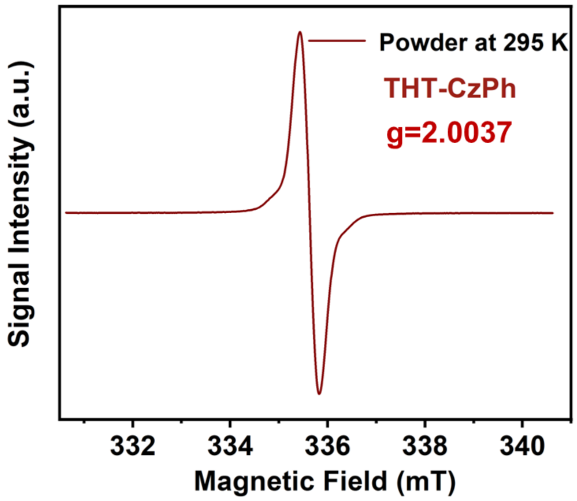

Fig. S18 EPR spectrum of **THT-CzPh** powder at 295 K.

S4.2 Photophysical properties of TT-CzPh and THT-CzPh

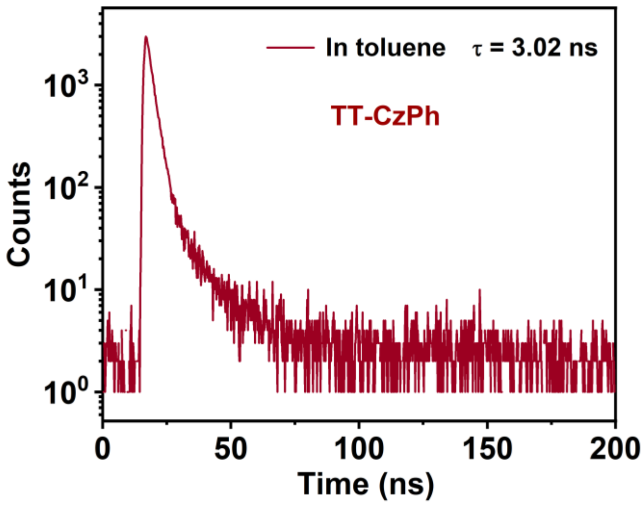

Fig. S19 Transient PL decay spectra (excitation at 370 nm) of **TT-CzPh** in toluene (10^{-5} M) at room temperature.

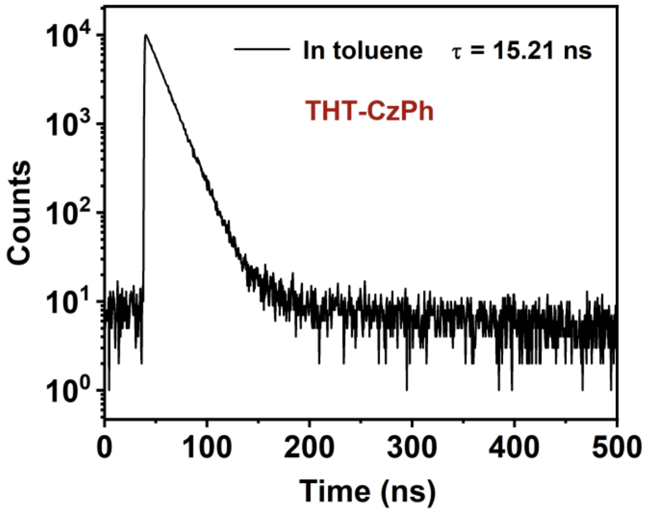

Fig. S20 Transient PL decay spectra (excitation at 370 nm) of **THT-CzPh** in toluene (10^{-5} M) at room temperature.

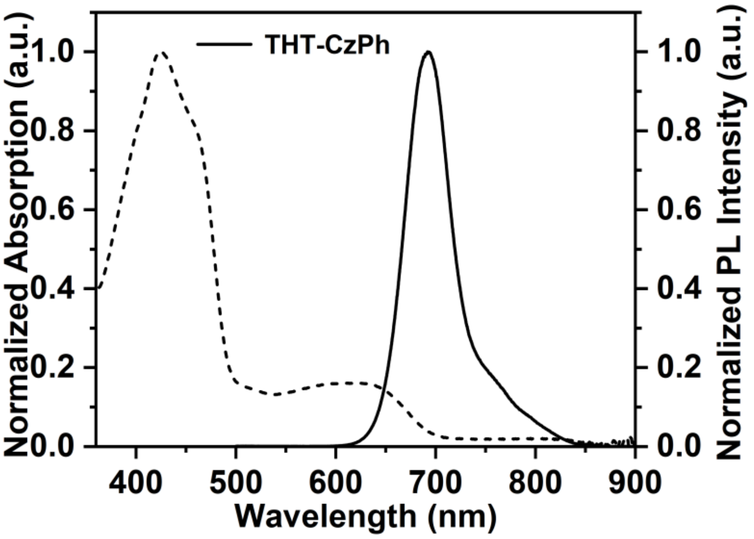

Fig. S21 Normalized absorption and emission spectra of **THT-CzPh** in toluene (10^{-5} M) at room temperature.

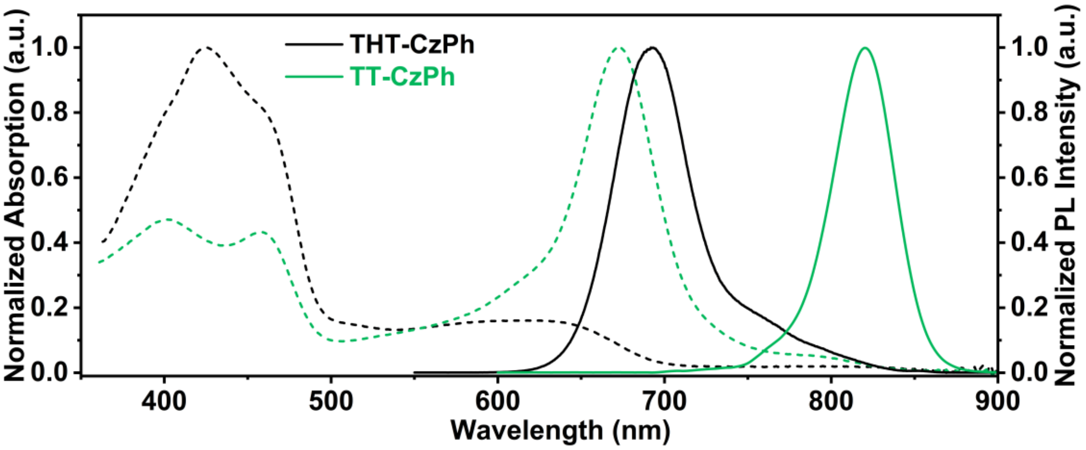

Fig. S22 Normalized absorption and emission spectra of **THT-CzPh** and **TT-CzPh** in toluene (10^{-5} M) at room temperature.

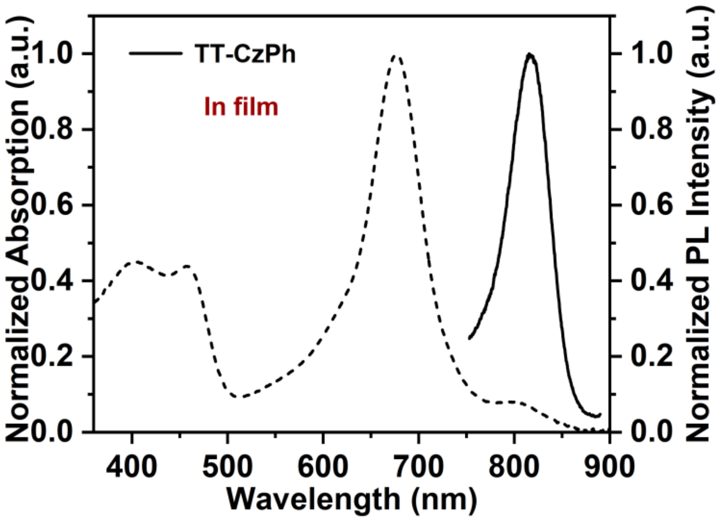

Fig. S23 Normalized absorption and emission spectra of **TT-CzPh** in PMMA film (1 wt% radical) at room temperature.

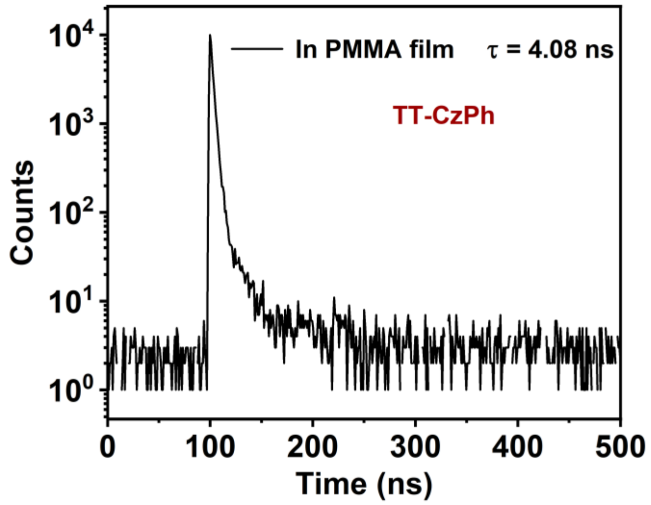

Fig. S24 Transient PL decay spectra (excitation at 370 nm) of **TT-CzPh** in PMMA film (10^{-5} M) at room temperature.

Table S1. Photophysical properties of **THT-CzPh** and **TT-CzPh** in toluene (10^{-5} M), **TT-CzPh** in PMMA film (1wt% radical) at room temperature.

Radical	Solvent	λ_{abs} (nm)	λ_{PL} (nm)	Φ_{PL}(%)	τ (ns)
THT-CzPh	Toluene	620	693	16.57	15.21
TT-CzPh	Toluene	785	822	5.20	3.02
	PMMA film	800	821	6.40	4.08

Notes: Measuring the fluorescence properties of radical compounds in the powder state often suffers from severe signal quenching due to strong aggregation-caused quenching (ACQ) effects. To accurately investigate the intrinsic solid-state photophysical properties of the luminescent radical in this study, we embedded the compounds into poly (methyl methacrylate) (PMMA) films at a low concentration (1 wt%). The PMMA matrix acts as an inert medium that physically isolates individual radical molecules, effectively minimizing aggregation and suppressing the ACQ effect, as well as reducing intermolecular interactions.

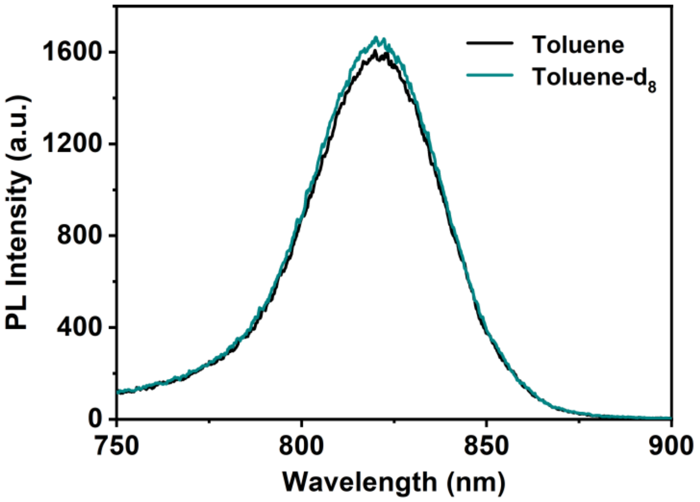

Fig. S25 PL spectra of TT-CzPh (10^{-5} M) in Toluene and Toluene-d_8 at room temperature.

Table S2. Photophysical properties of TT-CzPh (10^{-5} M) in Toluene and Toluene- d_8 at room temperature.

Radical	Solvent	λ_{abs} (nm)	λ_{PL} (nm)	Φ_{PL} (%)
TT-CzPh	Toluene	785	822	5.18
	Toluene-d_8	785	822	5.38

Notes: To evaluate the possible energy loss via vibrational energy transfer to the solvent, we measured the photoluminescence (PL) spectra and photoluminescence quantum

yields (PLQYs) of the compound in both toluene and deuterated toluene (toluene-d_8) under identical conditions. The PLQY in toluene was determined to be 5.18%, while a slightly enhanced PLQY of 5.38% was observed in deuterated toluene. Moreover, the PL intensity in toluene-d8 was also moderately increased, as shown in Fig. S24.

This marginal enhancement in PLQY and PL intensity is consistent with a minor reduction in non-radiative decay pathways due to weaker vibrational coupling in deuterated solvents. However, the relatively small difference suggests that the energy transfer from the excited state to solvent vibrational modes plays a limited role in the overall photophysical behavior of the compound in solution.

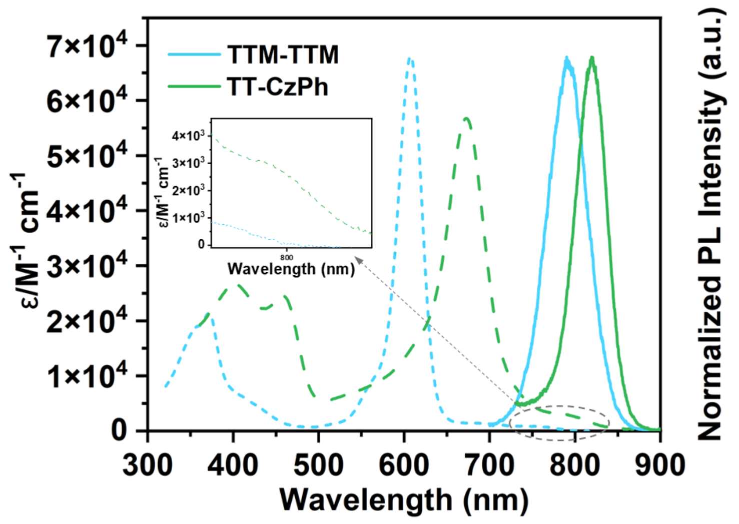

Fig. S26 Molar absorptivity (ε) of **TT-CzPh** (green) and **TTM-TTM** (blue) in toluene at room temperature.

S4.3 Theoretical calculations of radicals

All the calculations were performed using the Gaussian 16 program[5]. The geometry optimizations of all the radicals were performed at the theoretical level of (U) B3LYP/6-31G (d,p). With regard to the diradical TT-CzPh, the spin-unrestricted broken-symmetry wavefunction was used to characterize the open-shell singlet (OS) state of TT-CzPh diradical. The singlet-triplet energy gap ($\Delta E_{S\text{-}T}$) was calculated according to the following equation:

$$\triangle E_{S-T} = \left(E_{OS} - E_{T_1}\right)\frac{\langle S^2\rangle_{T_1}}{\langle S^2\rangle_{T_1} - \langle S^2\rangle_{OS}} \qquad (1)$$

where E_{OS} and E_{T1} correspond to the OS and T_1 state energies, and $\langle S^2\rangle_{OS}$ and $\langle S^2\rangle_{T}$ mean the spin contamination values of OS and T_1 states, respectively. While $\Delta E_{OS\text{-}CS}$ is calculated based on the energy difference between OS and CS state.

The diradical character y_0 is calculated by Yamaguchi's equation[6] at PUB3LYP/6-31G (d,p) level of theory and basis set:

$$y_0 = 1 - \frac{2T_0}{1 + T_0^2} \qquad (2)$$

$$T_0 = \frac{n_{HONO} - n_{LUNO}}{2} \qquad (3)$$

where, T_0 corresponds to the overlap between the two corresponding orbitals; n_{HONO} and n_{LUNO} are the occupation numbers of the highest occupied natural orbital and the lowest unoccupied natural orbital, respectively.

Table S3. Diradical Properties: y_0, $\Delta E_{S\text{-}T}$ and $\Delta E_{OS\text{-}CS}$ of **TTM-TTM** and **TT-CzPh**

**TT-CzPh**	E_{CS}(Hartree)	-9969.340442
	E_{OS}(Hartree)	-9969.355040
	E_{T1}(Hartree)	-9969.354149
	$<S^2>_{OS}$	1.03
	$<S^2>_{T1}$	2.04
	$\Delta E_{S\text{-}T}$ (kcal/mol)	-1.13
	$\Delta E_{OS\text{-}CS}$ (kcal/mol)	-9.16
	y_0	0.716
**TTM-TTM**	E_{CS}(Hartree)	-8818.312925
	E_{OS}(Hartree)	-8818.327657
	E_{T1}(Hartree)	-8818.326812
	$<S^2>_{OS}$	1.03
	$<S^2>_{T1}$	2.04
	$\Delta E_{S\text{-}T}$ (kcal/mol)	-1.07
	$\Delta E_{OS\text{-}CS}$ (kcal/mol)	-9.24
	y_0	0.735

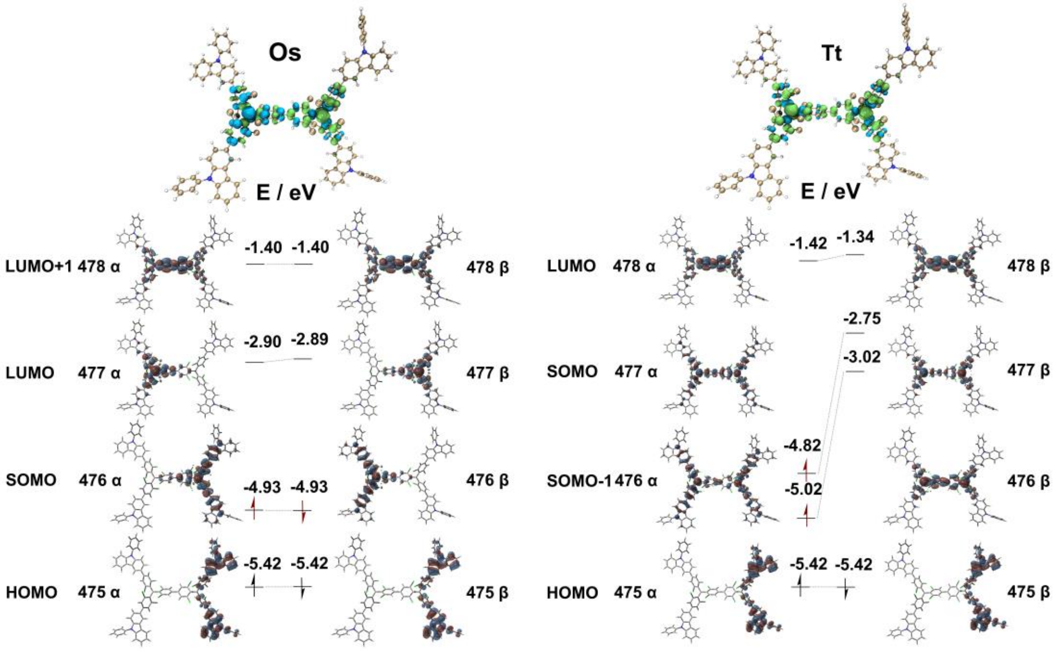

Fig. S27 Spin density distributions (isovalue = 0.002 e bohr^{-3}) (up) and frontier orbitals (down) of **TT-CzPh** in open-shell singlet (OS) ground state (left) and thermally excited triplet (T_t) state (right) calculated at the theoretical level of UB3LYP/6-31G (d,p).

Table S4. Summary of selected calculated excitation energies, oscillator strength and contributions of major electronic transitions of **TT-CzPh** and **TTM-TTM**

	**Transition**	**Wavelength (nm)**	**Energy (eV)**	**Osc.Strength (*f*)**	**Major Contribution**
**TT-CzPh**	OS→S	736.81	1.6827	0.0030	476A→ 477 A (or 476B→ 477 B)
		418.42	2.9631	0.4543	476A→ 478 A (or 476B→ 478 B)
		405.07	3.0608	0.3158	476A→ 479 A (or 476B→ 479 B)
	T_n→T	612.75	2.0234	0.0871	475 B →476 B
		435.98	2.8438	0.5722	477 A →478 A
		389.03	3.1870	0.1406	477 A →479 A
**TTM-TTM**	OS→S	681.36	1.8196	0.0000	256 A→ 257 A (or 256 B→ 257 B)
		397.50	3.1191	0.6110	256 A→ 258 A (or 256 B→ 258 B)
		372.15	3.3316	0.2275	256 A→ 259 A (or 256 B→ 259 B)
	T_n→T	513.72	2.8418	0.0492	255 B →256 B
		420.66	2.9474	0.4634	257 A →258 A
		376.18	3.2959	0.1519	257 A →260 A

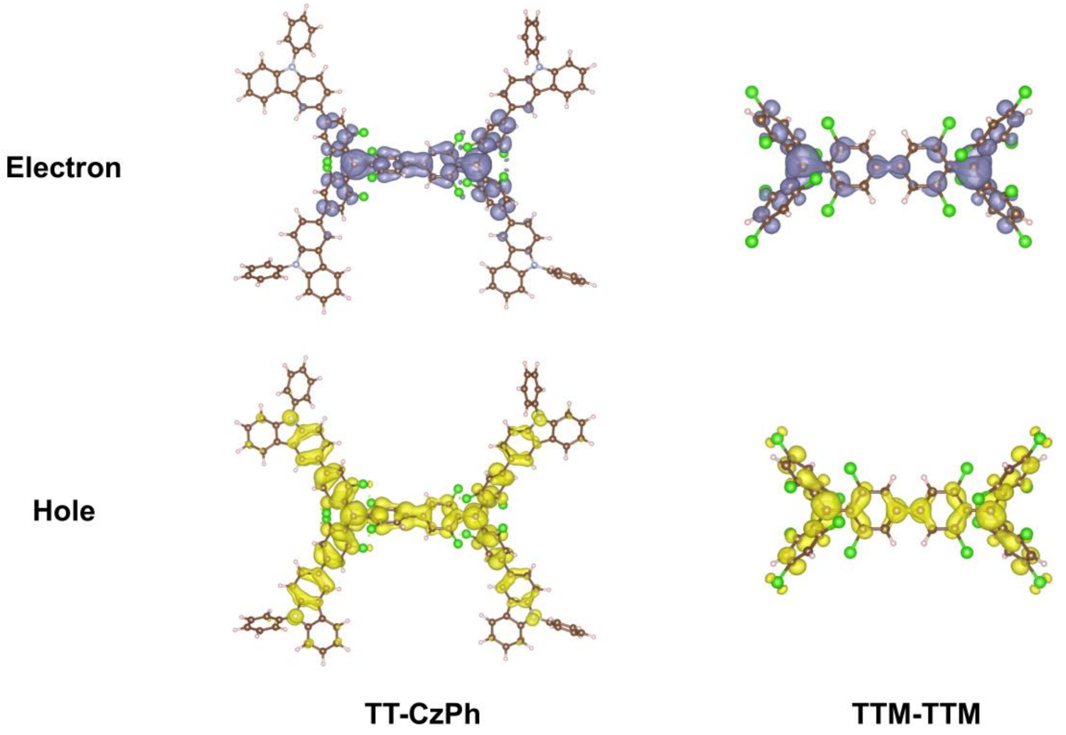

Fig. S28 Hole-electron analysis of the lower charge transfer state transition based on OS state of **TT-CzPh** and **TTM-TTM** (isovalue = 0.001 a.u.).

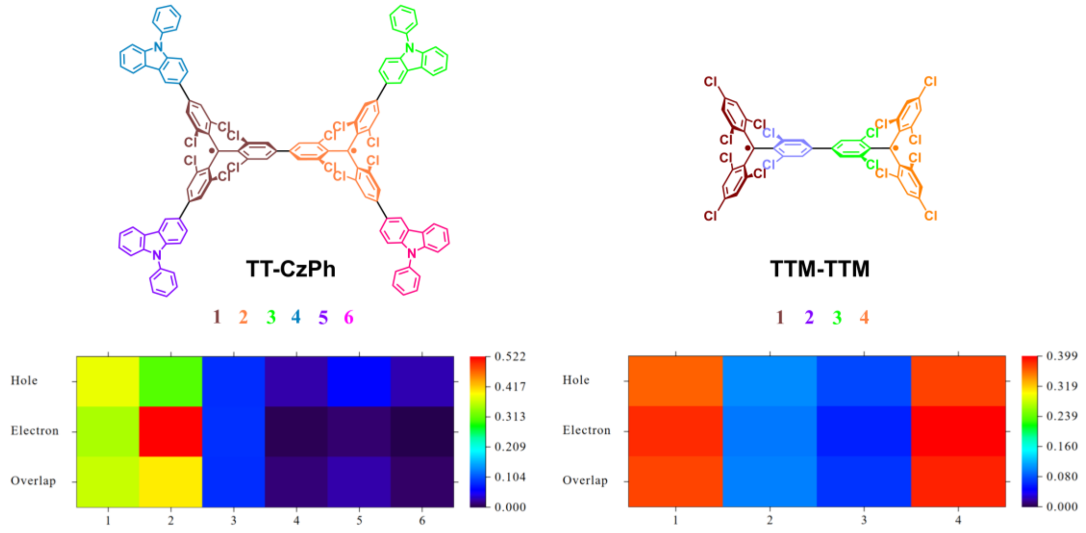

Fig. S29 The fragment definition (up penals) and heat map of the second excited state transition based on OS state (down panels) of **TT-CzPh** (left) and **TTM-TTM** (right).

Table S5. The relevant index of low-lying charge transfer state of **TT-CzPh** and **TTM-TTM** based on electron-hole analysis.

	**Sr (a.u.)**	**D (Å)**	**t (Å)**	**Excitation energy (eV)**	***f***
TT-CzPh	0.865	2.116	-3.914	1.769	0.0871
TTM-TTM	0.957	0.001	-5.216	1.948	0.0492

S4.4 Photothermal performance of radicals

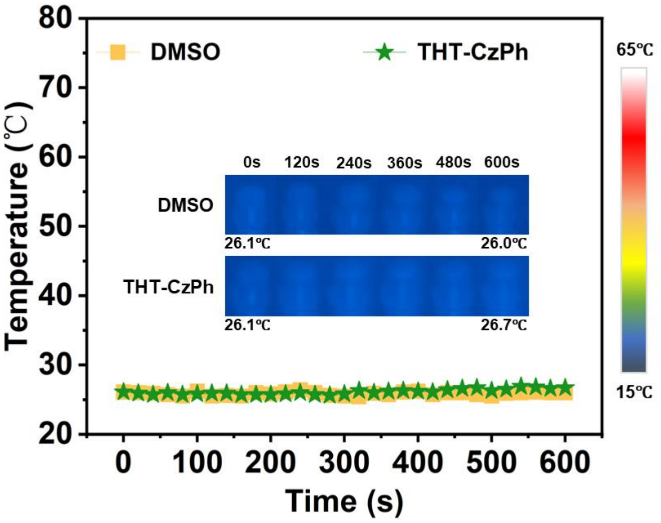

Fig. S30 The temperature variation curves of solvent DMSO and monoradical THT-CzPh (concentration: 25 μg mL^{-1}) under 655 nm laser irradiation and 1.0 W cm^{-2} power density. The temperature changes slightly within 10 min.

Notes: DMSO was selected for PCE measurements owing to its high boiling point (189 °C), excellent thermal stability, and low volatility, which collectively minimize solvent evaporation and ensure reliable photothermal performance under continuous laser irradiation.

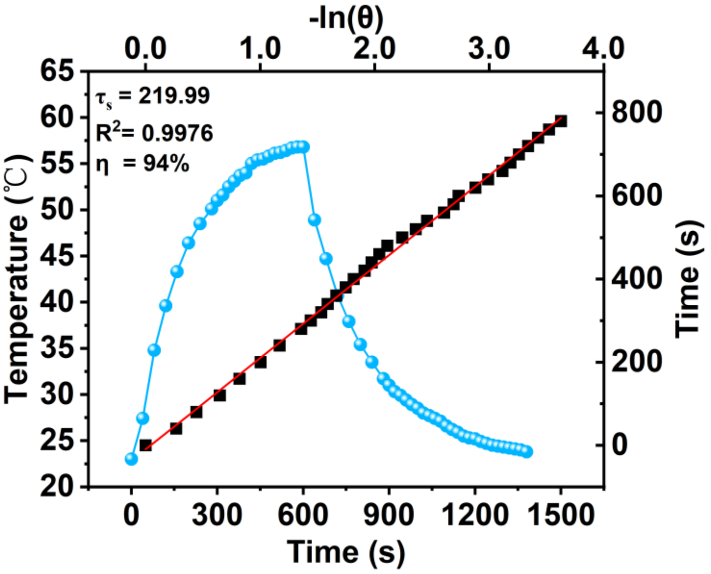

Fig. S31 Photothermal heating and cooling curves (linear fit-ln(θ)-cooling time) for TTM-TTM. (Solvent: DMSO)

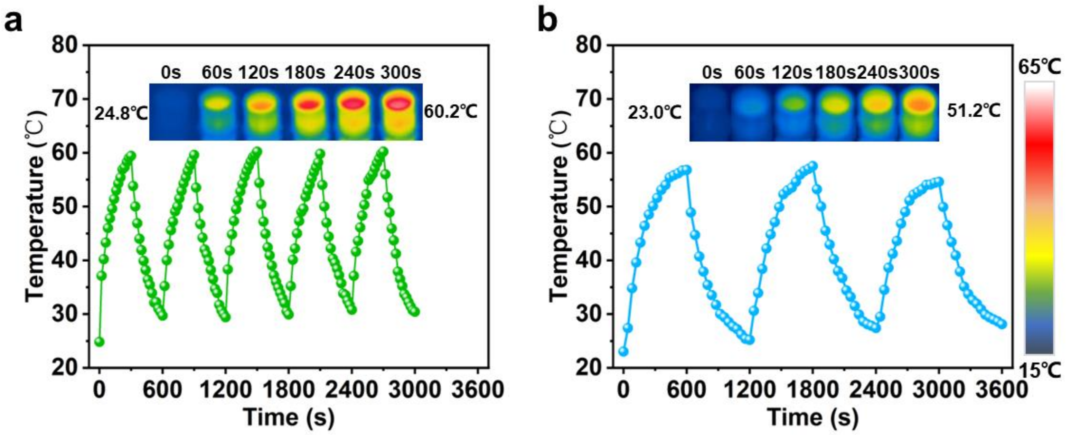

Fig. S32 Heating/cooling cycle profiles of TT-CzPh and TTM-TTM for the same time under 655 nm laser irradiation (1.0 W cm^{-2}). **a** Photothermal stability of TT-CzPh after five on/off cycles. **b** Photothermal stability of TTM-TTM after three on/off cycles. (Solvent: DMSO)

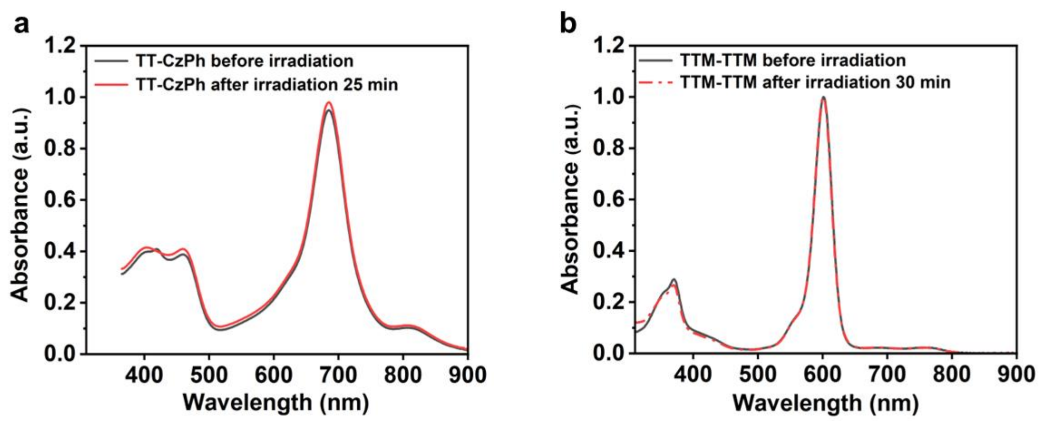

Fig. S33 UV/Vis absorption spectra of TT-CzPh **a** and TTM-TTM **b** in DMSO (concentration: 25 μg mL^{-1}) before and after 655 nm laser irradiation. (1.0 W cm^{-2})

S4.5 The particle size of TTM-TTM NPs

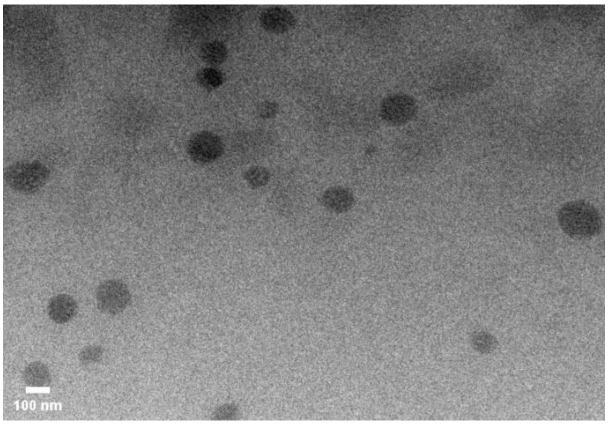

Fig. S34 TEM image of TTM-TTM NPs.

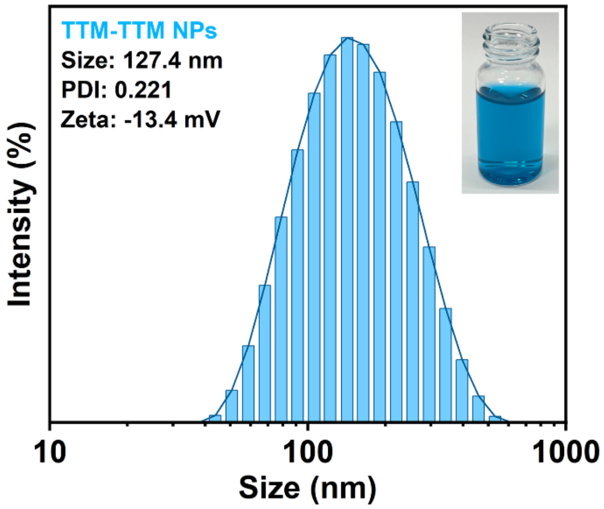

Fig. S35 The particle size distributions of TTM-TTM NPs in aqueous solution were determined by DLS method.

S4.6 EPR spectra of NPs

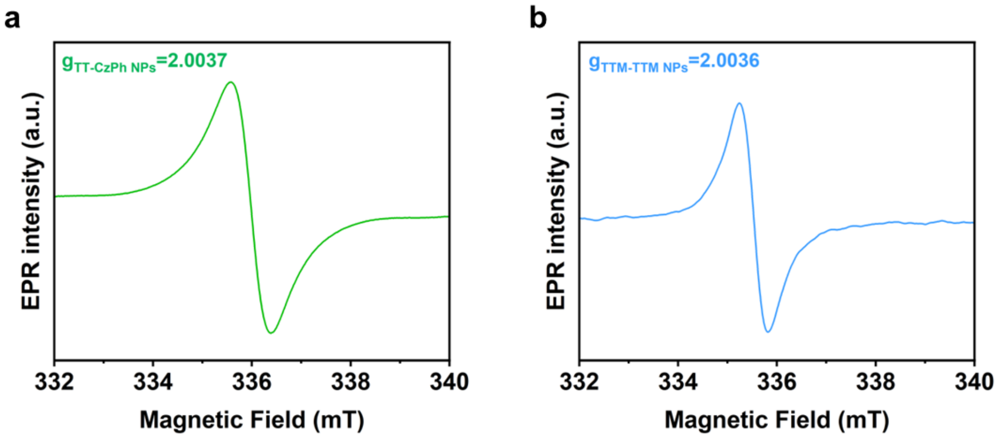

Fig. S36 EPR spectra of TT-CzPh NPs **a** and TTM-TTM NPs **b**.

S4.7 Normalized absorption and PL spectra of TT-CzPh NPs and TTM-TTM NPs

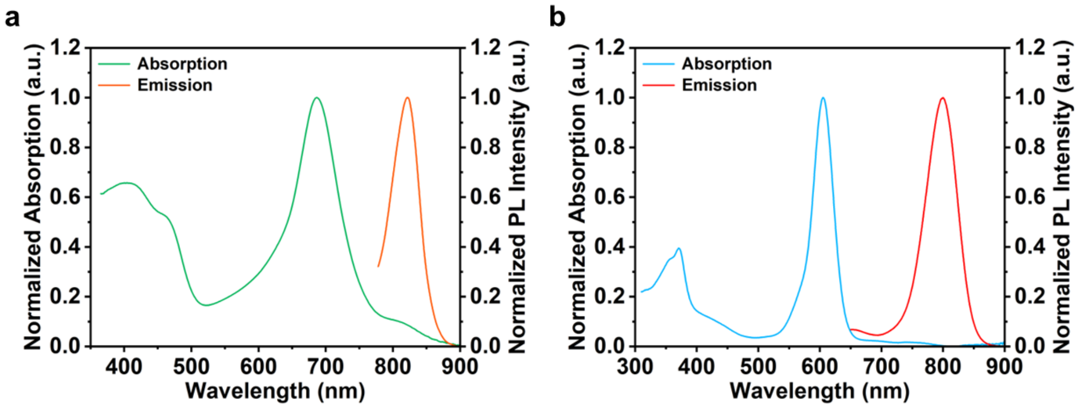

Fig. S37 UV/vis absorption and PL spectra of TT-CzPh NPs **a** (λ_{abs} = 687 nm, λ_{em} = 822 nm) and TTM-TTM NPs **b** (λ_{abs} = 605 nm, λ_{em} = 800 nm) in aqueous solution. (λ_{ex} = 655 nm)

S4.8 Image of NPs taken by smartphone

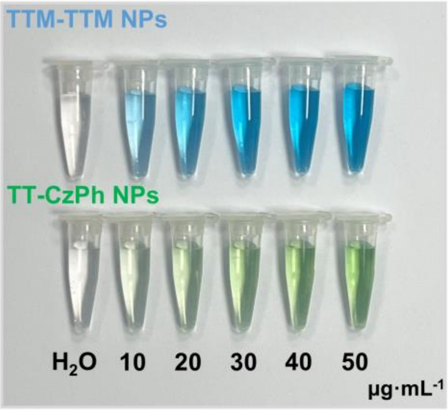

Fig. S38 The corresponding smartphone-shot image of TTM-TTM NPs and TT-CzPh NPs at different concentrations.

S4.9 Photothermal performance of TTM-TTM NPs

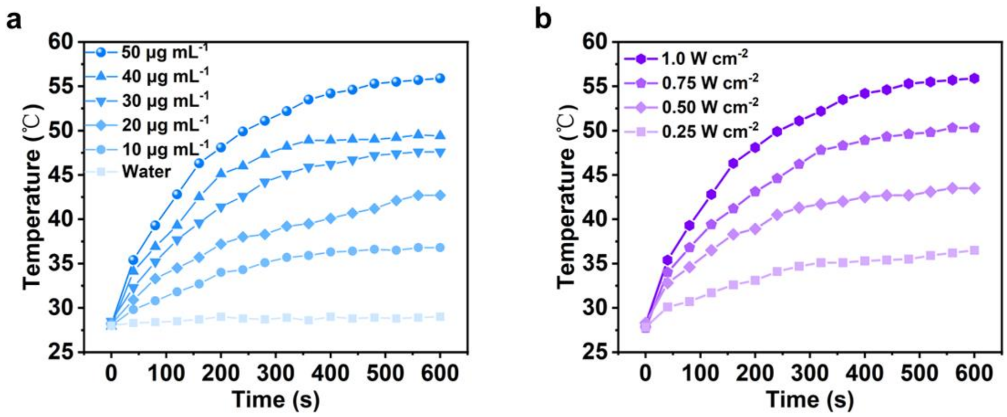

Fig. S39 **a** Concentration-dependent temperature variation of TTM-TTM NPs in aqueous solution under 655 nm laser irradiation (1.0 W cm^{-2}). **b** Power-density-dependent temperature variation of TTM-TTM NPs aqueous solution (50 μg mL^{-1}) under 655 nm laser irradiation.

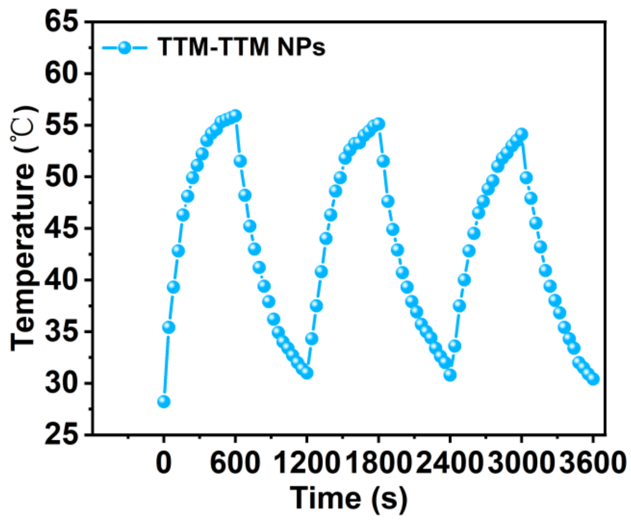

Fig. S40 Photothermal stability of TTM-TTM NPs after three on/off cycles of 655 nm laser irradiation (1.0 W cm^{-2}).

S4.10 Photothermal capacity of TT-CzPh NPs

Table S6. Photophysical properties of different types of photothermal therapeutic agents previously reported.

Name	λ_{abs} (nm)	ε_{max} (M^{-1} cm^{-1})	λ_{em} (nm)	PCE (%)	Ref.
TT-CzPh NPs	687	4.49×10^4	822	82	This work
2PhNVDPP NPs	703	3.03×10^4	1013	53	7
BOD NPs	391/514/742	9.30×10^4	-	71	8
$(NDI\text{-}2CB[7])^{\bullet-}$	1104	5.52×10^3	-	66.9	9
TTB-2 NPs	950	-	-	87.7	10
Na-NPs	727, 812	-	-	71.8	11
2F NPs	808	-	-	50	12
TPA-TBT NPs	620	-	830	44	13
AuNPs Aggregates	550	-	-	53.7	14
Pt@V_2C MXene		-	-	59.6	15
$Cu_{2-x}S$ NCs	550	-	680	50.6	16

S4.11 Photothermal stability of TT-CzPh NPs

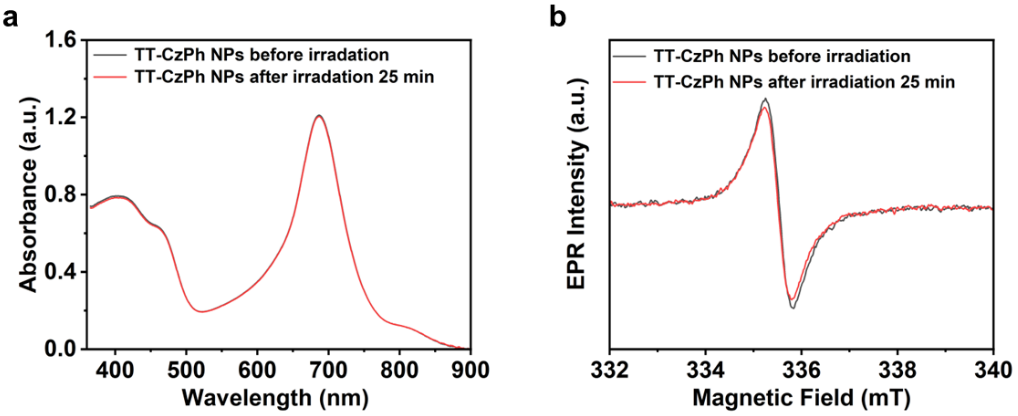

Fig. S41 a UV/Vis absorption spectra of TT-CzPh NPs before and after irradiation. **b** EPR spectra of TT-CzPh NPs of TT-CzPh NPs before and after irradiation.

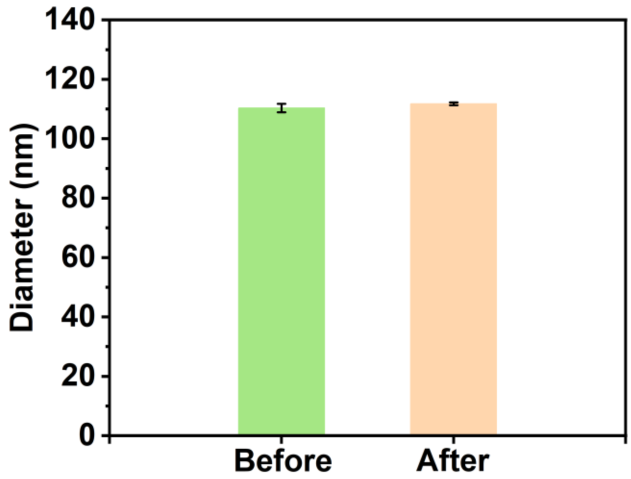

Fig. S42 Size of TT-CzPh NPs before and after irradiation.

S4.12 Stability of TT-CzPh NPs over time

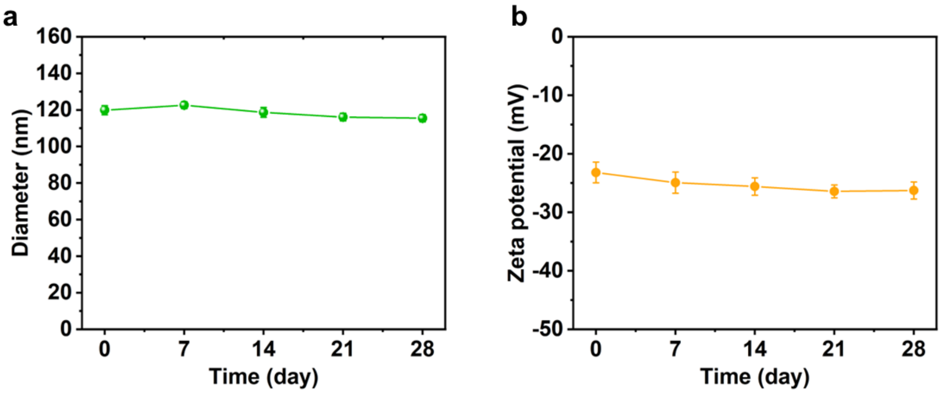

Fig. S43 a Size change of TT-CzPh NPs after 28 days of storage. **b** Zeta-potentials change of TT-CzPh NPs after 28 days of storage.

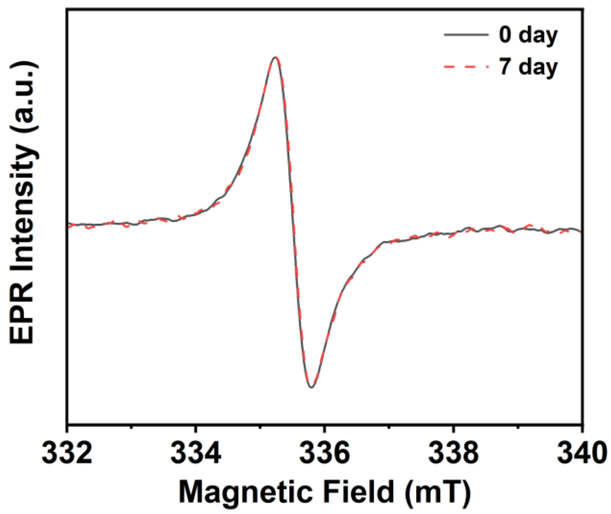

Fig. S44 EPR spectra of TT-CzPh NPs after 7 days of storage.

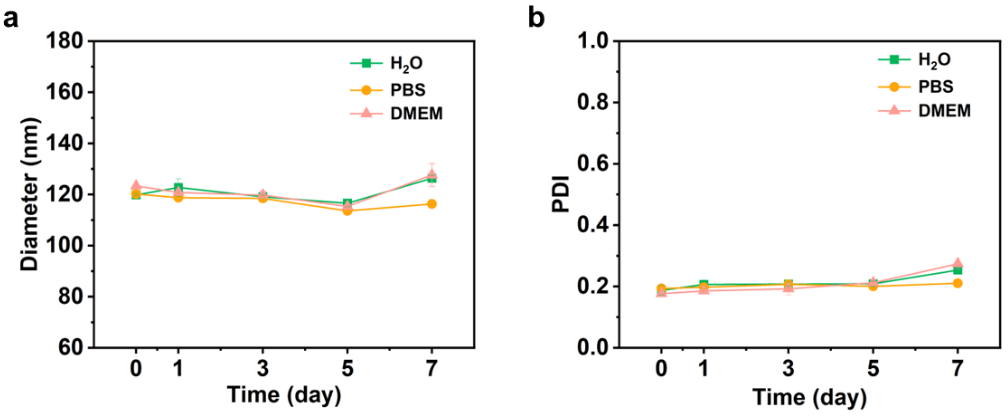

Fig. S45 Size **a** and PDI **b** changes of TT-CzPh NPs in solutions containing H_2O, PBS and DMEM.

S4.13 In vitro cellular experiments with TT-CzPh NPs

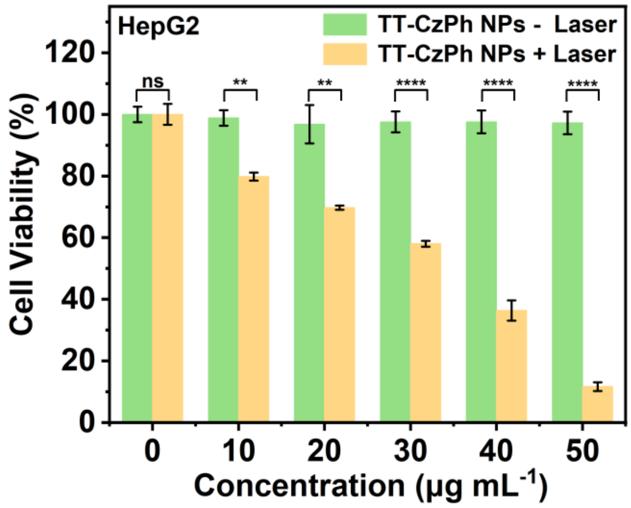

Fig. S46 In vitro photothermic ablation of HepG2 cells after being treated with TT-CzPh NPs with or without 655 nm irradiation (1.0 W cm $^{-2}$) for 3 min (n=3, *P$<$0.05, **P$<$0.01, ***P$<$0.001, ****P$<$0.0001).

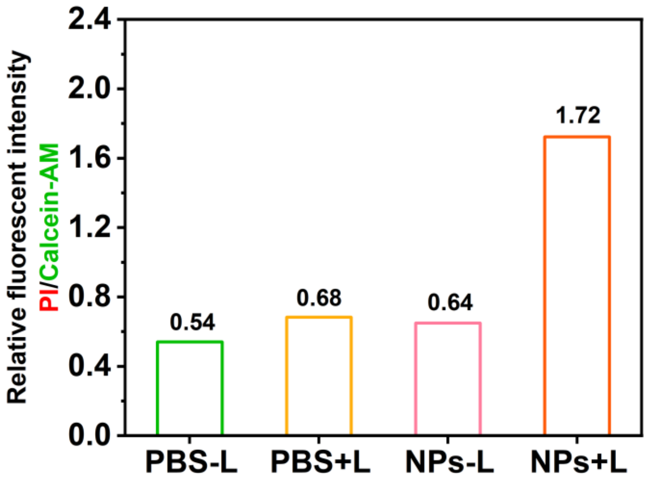

Fig. S47 The ratio metric image of PI (Red) /Calcein (Green) value after different treatment.

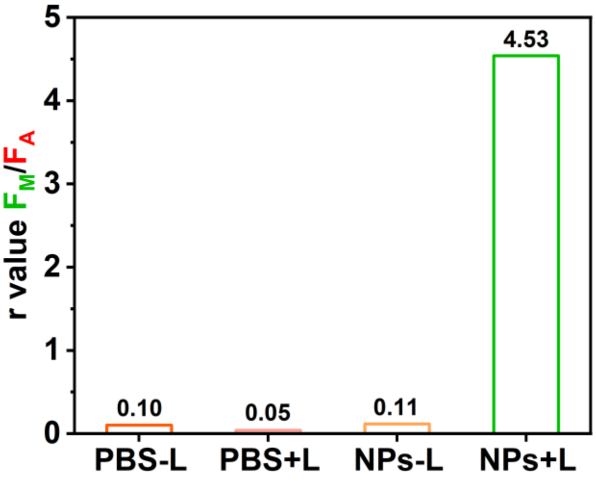

Fig. S48 The ratio metric image of JC-1 Monomer/Aggregate value after different treatment.

S4.14 Fluorescence stability of TT-CzPh NPs

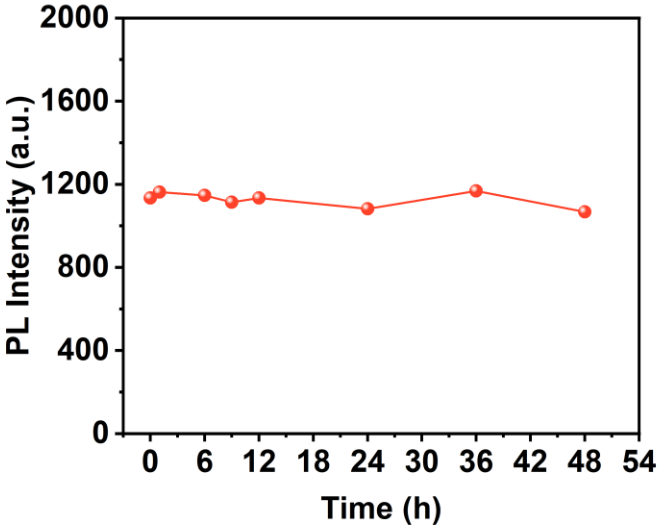

Fig. S49 Relationship between the fluorescence intensity and time of TT-CzPh NPs after laser irradiation (λ_{ex} = 655 nm, λ_{em} = 822 nm).

S4.15 In vivo experiments with TT-CzPh NPs

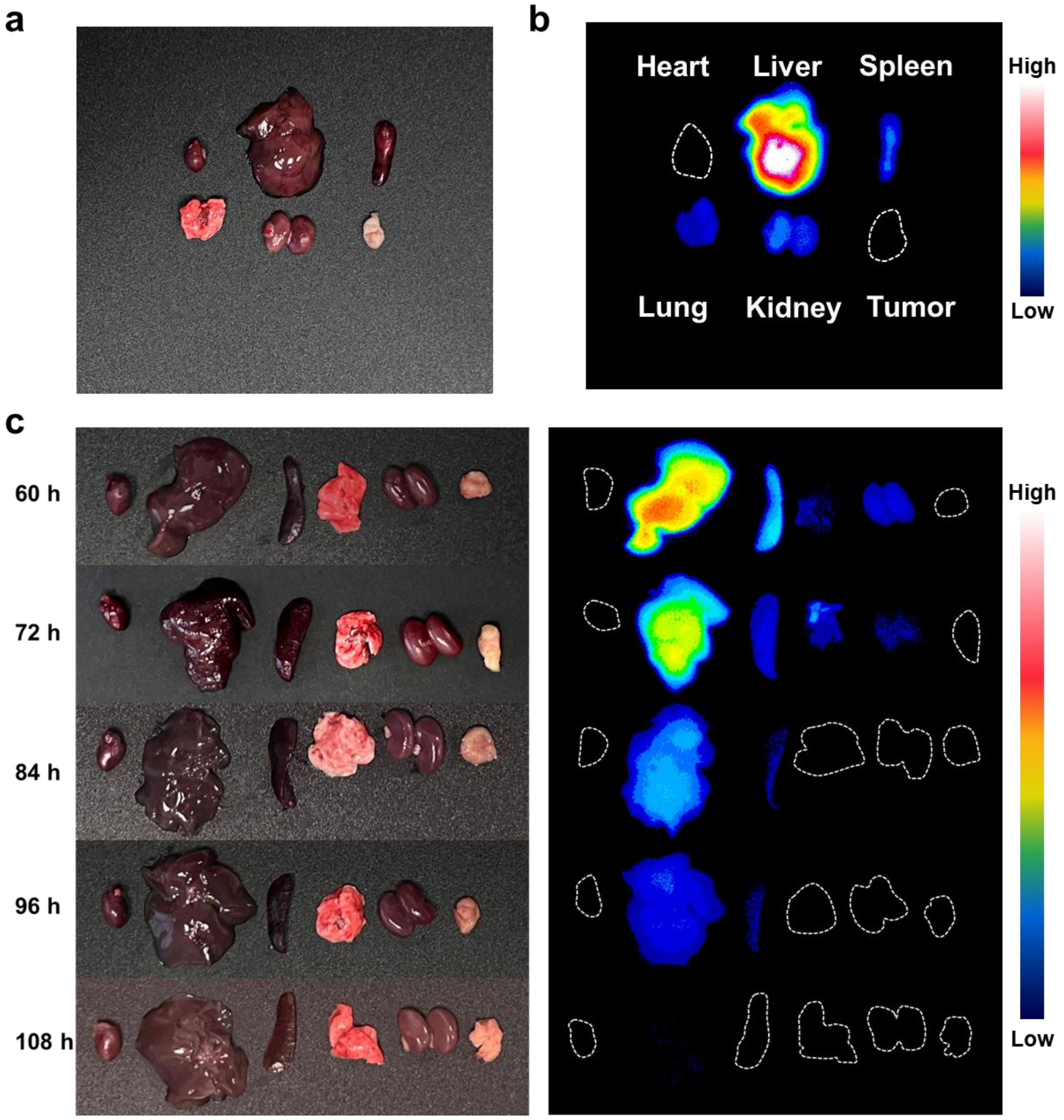

Fig. S50 At 48 h post-injection of TT-CzPh NPs, ex vivo bright-field images **a** and NIR fluorescence images **b** of major organs and tumor. **c** shows the bright-field images (left) and NIR fluorescence images (right) of major organs and tumor at different time points 48 h later.

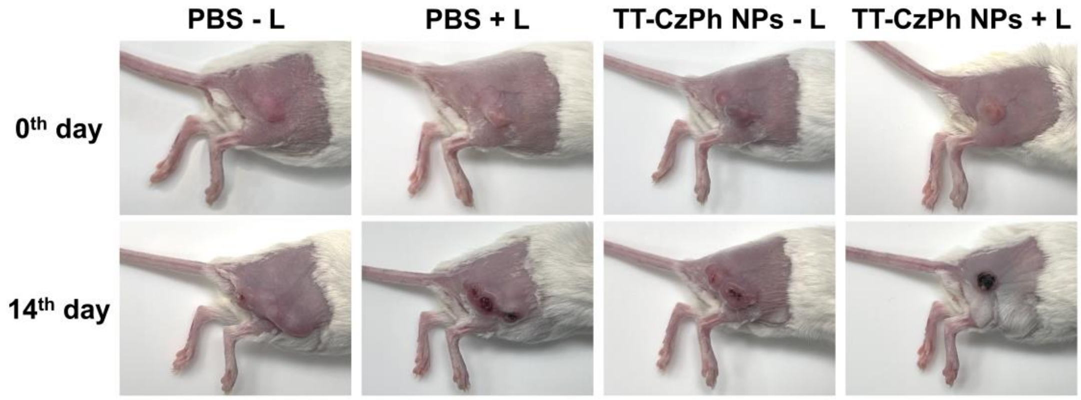

Fig. S51 Photographs of mice were taken at day 0 and day 14 to document the effects of different treatments.

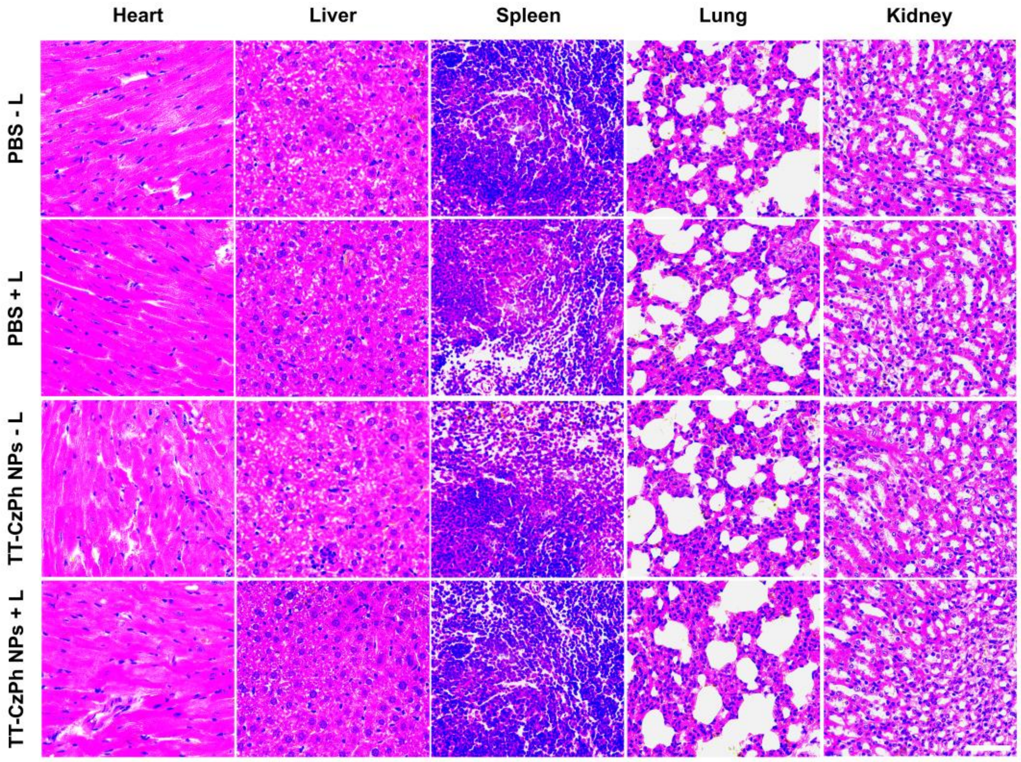

Fig. S52 H&E staining images of major organs (heart, liver, spleen, lung, kidney) from mice treated with PBS - L, PBS + L, TT-CzPh NPs - L, and TT-CzPh NPs + L. Scale bar = 50 μm.

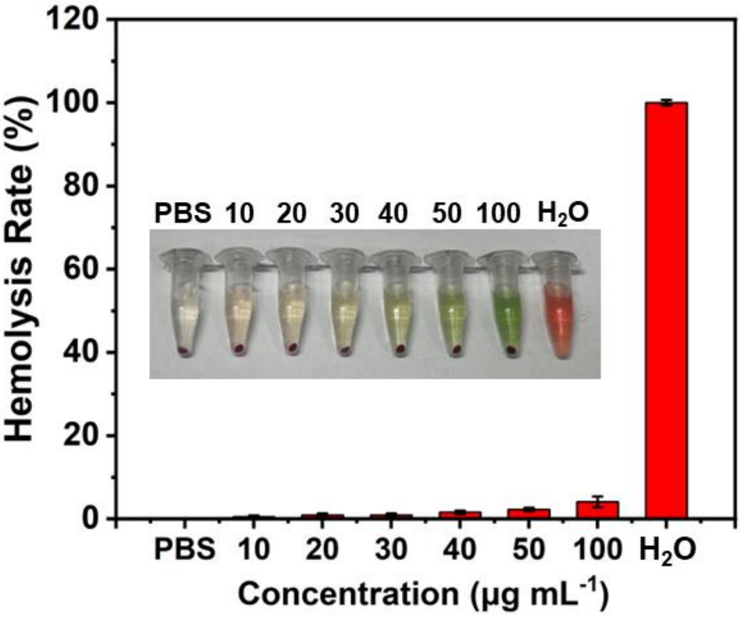

Fig. S53 Relative hemolysis rate of red blood cells treated with different concentrations of TT-CzPh NPs by using water as a positive control and PBS as a negative control.

S5. Detailed data of TT-CzPh excited states calculated by TD-DFT

OS→S_1: at TD-UB3LYP/6-31G (d,p) level of theory

Excited State 1: 1.497-A 1.6827 eV 736.81 nm f=0.0030 <S**2>=0.311
476A -> 477A 0.75508
476B -> 477B 0.61780
This state for optimization and/or second-order correction.
Total Energy, E (TD-HF/TD-DFT) = -9969.29320066
Copying the excited state density for this state as the 1-particle RhoCI density.

Excited State 2: 1.439-A 1.7693 eV 700.74 nm f=0.3310 <S**2>=0.268
476A -> 477A -0.62782
476B -> 477B 0.76381

Excited State 3: 2.363-A 2.0653 eV 600.33 nm f=0.1226 <S**2>=1.146
466A -> 477A -0.19252
471A -> 477A 0.11262
474A -> 477A 0.92888
475B -> 477B -0.16113
476B -> 479B -0.11755

Excited State 4: 2.366-A 2.0723 eV 598.29 nm f=0.2187 <S**2>=1.149
474A -> 477A 0.16094
476A -> 479A 0.12048
466B -> 477B 0.19116
471B -> 477B 0.10935
475B -> 477B 0.92928

Excited State 5: 2.376-A 2.1422 eV 578.78 nm f=0.1260 <S**2>=1.162
467A -> 477A -0.11475
473A -> 477A 0.79315
473B -> 477B -0.51748

Excited State 6: 2.331-A 2.1624 eV 573.36 nm f=0.0059 <S**2>=1.108
473A -> 477A 0.52124
473B -> 477B 0.79516

Excited State 7: 2.307-A 2.3311 eV 531.86 nm f=0.0069 <S**2>=1.081
475A -> 477A 0.99370

Excited State 8: 2.309-A 2.3437 eV 529.02 nm f=0.0067 <S**2>=1.083
474B -> 477B 0.99456

Excited State 9: 2.368-A 2.4225 eV 511.81 nm f=0.0006 <S**2>=1.151
467A -> 477A 0.21644
472A -> 477A 0.67651
476A -> 477A -0.13968
476A -> 478A 0.13504
467B -> 477B 0.18630
472B -> 477B 0.54530
476B -> 477B -0.12932
476B -> 478B 0.13787

Excited State 10: 2.217-A 2.4874 eV 498.44 nm f=0.0645 <S**2>=0.979
467A -> 477A -0.11588
472A -> 477A -0.60975
467B -> 477B 0.15256
472B -> 477B 0.73297

Excited State 11: 2.364-A 2.5765 eV 481.21 nm f=0.0009 <S**2>=1.147
466A -> 477A -0.22222
469A -> 477A -0.15577
471A -> 477A 0.89753
474A -> 477A -0.21115
476B -> 479B -0.14748

Excited State 12: 2.366-A 2.5882 eV 479.04 nm f=0.0005 <S**2>=1.150
476A -> 479A 0.15064
466B -> 477B 0.21722
469B -> 477B -0.15258
471B -> 477B 0.89781
475B -> 477B -0.20920

Excited State 13: 2.359-A 2.5974 eV 477.34 nm f=0.0001 <S**2>=1.141
467A -> 477A -0.20731
469A -> 477A 0.82321
470A -> 477A -0.24790
471A -> 477A 0.15752
472A -> 477A 0.20986
473A -> 477A -0.18590
469B -> 477B -0.10963
476B -> 478B -0.14767

Excited State 14: 2.344-A 2.6077 eV 475.45 nm f=0.0001 <S**2>=1.124
469A -> 477A 0.11734
476A -> 478A -0.15602

467B -> 477B -0.21152
469B -> 477B 0.83679
470B -> 477B -0.18907
471B -> 477B 0.15072
472B -> 477B 0.20453
473B -> 477B -0.18856

Excited State 15: 2.403-A 2.6954 eV 459.98 nm f=0.0004 <S**2>=1.194
457A -> 477A -0.11928
464A -> 477A -0.18635
467A -> 477A 0.45477
468A -> 477A -0.29762
469A -> 477A 0.24875
471A -> 477A 0.10928
472A -> 477A -0.29361
476A -> 478A 0.19770
464B -> 477B 0.10051
467B -> 477B 0.29997
468B -> 477B -0.21775
469B -> 477B 0.17440
472B -> 477B -0.25723
476B -> 478B 0.27952

Excited State 16: 2.407-A 2.7071 eV 458.00 nm f=0.0202 <S**2>=1.199
464A -> 477A 0.20328
467A -> 477A -0.36290
468A -> 477A 0.14030
469A -> 477A -0.20514
476A -> 478A 0.31902
457B -> 477B -0.12985
464B -> 477B 0.23330
467B -> 477B 0.48799
468B -> 477B -0.22605
469B -> 477B 0.29038
471B -> 477B 0.14012
472B -> 477B -0.13708
476B -> 478B -0.22604

Excited State 17: 2.463-A 2.7737 eV 446.99 nm f=0.0474 <S**2>=1.266
463A -> 477A -0.18985
464A -> 477A 0.11368
466A -> 477A 0.75386
469A -> 477A -0.14233
471A -> 477A 0.28003

474A -> 477A 0.17601
476B -> 479B 0.34550

Excited State 18: 2.478-A 2.7806 eV 445.89 nm f=0.0655 <S**2>=1.285
476A -> 479A 0.37404
463B -> 477B -0.16562
464B -> 477B 0.10790
466B -> 477B 0.73824
469B -> 477B 0.16215
471B -> 477B -0.28389
475B -> 477B -0.18326

Excited State 19: 2.348-A 2.8010 eV 442.65 nm f=0.0047 <S**2>=1.128
462A -> 477A 0.29570
464A -> 477A 0.56435
467A -> 477A -0.17605
468A -> 477A -0.21871
469A -> 477A 0.12007
470A -> 477A 0.40327
462B -> 477B 0.16813
464B -> 477B -0.36886
467B -> 477B -0.13752
468B -> 477B -0.10603
470B -> 477B 0.12421
476B -> 478B 0.18053

Excited State 20: 2.357-A 2.8073 eV 441.65 nm f=0.0287 <S**2>=1.138
462A -> 477A -0.20263
464A -> 477A -0.30346
469A -> 477A 0.17766
470A -> 477A 0.80322
468B -> 477B 0.11652
470B -> 477B -0.18856
476B -> 478B -0.24752

$T_t \rightarrow T_1$: at TD-UB3LYP/6-31G (d,p) level of theory
Excited State 1: 3.079-A 2.0234 eV 612.75 nm f=0.0871 <S**2>=2.121
466B -> 476B -0.12009
474B -> 476B 0.83729
474B -> 477B -0.36429
475B -> 476B -0.27139
This state for optimization and/or second-order correction.
Total Energy, E(TD-HF/TD-DFT) = -9969.27979026

Copying the excited state density for this state as the 1-particle RhoCI density.

Excited State 2: 3.080-A 2.0275 eV 611.50 nm f=0.2184 <S**2>=2.122
465B -> 476B -0.11749
474B -> 476B 0.27319
475B -> 476B 0.83091
475B -> 477B 0.37937

Excited State 3: 3.079-A 2.0858 eV 594.43 nm f=0.1664 <S**2>=2.120
467B -> 476B 0.18615
472B -> 476B -0.13040
472B -> 477B 0.33726
473B -> 476B 0.88112

Excited State 4: 3.074-A 2.1186 eV 585.22 nm f=0.0009 <S**2>=2.112
458B -> 476B 0.10716
472B -> 476B 0.89363
473B -> 476B 0.12565
473B -> 477B 0.35249

Excited State 5: 3.081-A 2.3819 eV 520.53 nm f=0.0231 <S**2>=2.123
470B -> 476B 0.11622
475B -> 476B -0.46378
475B -> 477B 0.83846

Excited State 6: 3.082-A 2.3900 eV 518.77 nm f=0.0220 <S**2>=2.125
471B -> 476B -0.13294
474B -> 476B 0.44953
474B -> 477B 0.84193

Excited State 7: 3.102-A 2.4401 eV 508.11 nm f=0.0024 <S**2>=2.155
477A -> 478A -0.17315
467B -> 476B 0.24285
467B -> 477B 0.10797
468B -> 476B 0.10828
469B -> 476B -0.14760
472B -> 476B -0.31501
472B -> 477B 0.28751
473B -> 476B -0.27969
473B -> 477B 0.72502

Excited State 8: 3.160-A 2.4427 eV 507.58 nm f=0.0008 <S**2>=2.246
477A -> 478A -0.33406
461B -> 476B 0.10192

467B -> 476B 0.46092
469B -> 476B -0.25928
472B -> 476B 0.25038
472B -> 477B 0.31696
473B -> 476B -0.28489
473B -> 477B -0.44918

Excited State 9: 3.131-A 2.4639 eV 503.21 nm f=0.0179 <S**2>=2.201
473A -> 478A -0.10187
477A -> 478A 0.29509
457B -> 476B -0.13964
467B -> 476B -0.34619
472B -> 477B 0.79718
473B -> 476B -0.18653

Excited State 10: 3.073-A 2.5346 eV 489.16 nm f=0.0010 <S**2>=2.110
469B -> 476B -0.13604
470B -> 476B 0.35262
471B -> 476B 0.79344
471B -> 477B -0.28684
474B -> 477B 0.26847

Excited State 11: 3.073-A 2.5414 eV 487.86 nm f=0.0009 <S**2>=2.111
469B -> 476B 0.15789
470B -> 476B 0.80275
470B -> 477B 0.29721
471B -> 476B -0.32960
471B -> 477B -0.10379
475B -> 477B -0.24938

Excited State 12: 3.073-A 2.5604 eV 484.24 nm f=0.0014 <S**2>=2.110
468B -> 476B 0.87054
469B -> 476B 0.14278
469B -> 477B -0.31582
473B -> 477B -0.24090

Excited State 13: 3.095-A 2.5755 eV 481.39 nm f=0.0344 <S**2>=2.144
477A -> 478A -0.23600
467B -> 476B 0.19728
468B -> 476B -0.15723
468B -> 477B -0.29665
469B -> 476B 0.82226
470B -> 476B -0.10424
471B -> 476B 0.17757

472B -> 477B 0.12949

Excited State 14: 3.100-A 2.7202 eV 455.78 nm f=0.0026 <S**2>=2.153
476A -> 478A -0.21669
456B -> 476B -0.14654
461B -> 477B -0.17078
464B -> 476B 0.63925
467B -> 477B 0.55063
468B -> 476B -0.20895
473B -> 477B -0.18707

Excited State 15: 3.128-A 2.7411 eV 452.32 nm f=0.0602 <S**2>=2.196
476A -> 479A 0.16283
477A -> 478A -0.21664
477A -> 479A -0.20607
460B -> 476B 0.10141
461B -> 476B 0.19124
464B -> 477B -0.16621
465B -> 476B 0.32520
465B -> 477B -0.33017
466B -> 476B 0.52213
466B -> 477B -0.21241
467B -> 476B -0.22694
469B -> 476B -0.12516
471B -> 476B 0.20869
474B -> 477B -0.17913

Excited State 16: 3.120-A 2.7461 eV 451.49 nm f=0.1961 <S**2>=2.184
477A -> 478A 0.38387
477A -> 480A -0.13647
461B -> 476B -0.32337
462B -> 476B -0.11344
464B -> 477B 0.29763
465B -> 476B 0.45739
466B -> 477B -0.29673
467B -> 476B 0.37852
470B -> 476B 0.14452
475B -> 477B 0.10918

Excited State 17: 3.131-A 2.7478 eV 451.21 nm f=0.1307 <S**2>=2.201
476A -> 480A 0.16523
477A -> 478A 0.28724
477A -> 480A 0.21290
461B -> 476B -0.24315

463B -> 476B -0.10594
464B -> 477B 0.21979
465B -> 476B -0.35354
465B -> 477B -0.27742
466B -> 476B 0.41768
466B -> 477B 0.23825
467B -> 476B 0.27673
470B -> 476B -0.21207
471B -> 476B 0.11650
475B -> 477B -0.17736

Excited State 18: 3.154-A 2.7896 eV 444.46 nm f=0.0333 <S**2>=2.237
476A -> 479A 0.22407
477A -> 479A -0.27870
462B -> 476B 0.51771
462B -> 477B -0.39037
463B -> 476B 0.47251
463B -> 477B -0.21084
471B -> 476B 0.16694
471B -> 477B 0.12212
474B -> 477B -0.13868

Excited State 19: 3.161-A 2.7938 eV 443.78 nm f=0.0515 <S**2>=2.249
476A -> 480A 0.22516
477A -> 480A 0.30049
462B -> 476B -0.43541
462B -> 477B -0.22106
463B -> 476B 0.50290
463B -> 477B 0.39346
465B -> 476B 0.12263
470B -> 476B -0.17083
470B -> 477B 0.13509
475B -> 477B -0.13987

Excited State 20: 3.159-A 2.8205 eV 439.58 nm f=0.0082 <S**2>=2.245
476A -> 478A 0.37284
477A -> 485A 0.16437
456B -> 476B 0.16977
458B -> 476B -0.33580
461B -> 477B -0.45166
464B -> 476B 0.46007
467B -> 477B -0.14612
468B -> 476B 0.19929
469B -> 477B 0.23646

473B -> 477B 0.14802

References

1. Peng, Q. M. et al. Organic light-emitting diodes using a neutral π radical as emitter: the emission from a doublet. *Angewandte Chemie-International Edition* **127**, 7197-7201 (2015).

2. Abdurahman, A. et al. Understanding the luminescent nature of organic radicals for efficient doublet emitters and pure-red light-emitting diodes. *Nature materials* **19**, 1224-1229 (2020).

3. Abdurahman, A. et al. A highly stable organic luminescent diradical. *Angewandte Chemie-International Edition* **62**, e202300772 (2023).

4. Ji, Y. et al. 77 % Photothermal conversion in blatter-type diradicals: photophysics and photodynamic applications. *Angewandte Chemie-International Edition* **62**, e202311387 (2023).

5. Frisch, M. J. et al. Gaussian 16. *Gaussian 16 Rev. C.01, Wallingford, CT.* (2016).

6. Yamaguchi, K. The electronic structures of biradicals in the unrestricted Hartree-Fock approximation. *Chemical Physics Letters* **33**, 330-335 (1975).

7. Feng, L. et al. Highly stable near-infrared II luminescent diradicaloids for cancer phototheranostics. *Journal of the American Chemical Society* **146**, 32582-32594 (2024).

8. Sun, T. T. et al. Rodlike nanomaterials from organic diradicaloid with high photothermal conversion capability for tumor treatment. *Aggregate* **4**, e362 (2023).

9. Zhou, Z. K. et al. A water-stable and red-emissive radical cation for Mutp53 cancer therapy. *Angewandte Chemie-International Edition* **61**, e202212671 (2022).

10. Gao, Y. J. et al. A stable open-shell conjugated diradical polymer with ultra-high photothermal conversion efficiency for NIR-II photo-immunotherapy of metastatic tumor. *Nano-Micro Letters* **16**, 21 (2024).

11. Gao, H. et al. Molecular engineering of corrole radicals by polycyclic aromatic fusion: towards open-shell near-infrared materials for efficient photothermal therapy. *Angewandte Chemie-International Edition* **62**, e202309208 (2023).

12. Lee, K.-W. et al. Non-fused conjugated oligomer with manipulated intermolecular charge transfer for highly efficient near-infrared phototheranostics of cancer. *Advanced Functional Materials* **35**, 2421765 (2025).

13. Xie, H. L. et al. Design of one-for-all near-infrared aggregation-induced emission nanoaggregates for boosting theranostic efficacy. *ACS Nano* **17**, 4591-4600 (2023).
14. Yan, X. et al. Bioorthogonal "Click and Release" reaction-triggered aggregation of gold nanoparticles combined with released lonidamine for enhanced cancer photothermal therapy. *Angewandte Chemie-International Edition* **63**, e202318539 (2024).
15. He, X. J. et al. Platinum nanoparticles regulated V_2C MXene nanoplatforms with NIR-II enhanced nanozyme effect for photothermal and chemodynamic anti-infective therapy. *Advanced Materials* **36**, 2400366 (2024).
16. Lee, I. et al. Penicillamine-induced decomposition of copper sulfide nanocrystals after microbial infection treatment. *Chemical Engineering Journal* **505**, 159195 (2025).
